# DNA-PAINT resolves E-cadherin-independent cross-junctional F-actin organization in *Drosophila* embryonic tissue

**DOI:** 10.1016/j.bpj.2026.01.026

**Published:** 2026-01-17

**Authors:** Matthias Häring, Yuanshu Zhang, Na Zhang, Edward S. Allgeyer, Jennifer H. Richens, George Sirinakis, Zhiyi Lv, Daniel St Johnston, Fred Wolf, Jörg Großhans, Deqing Kong

**Affiliations:** 1 Göttingen Campus Institute for Dynamics of Biological Networks (CIDBN), University of Göttingen, Göttingen, Germany; 2 Max Planck Institute for Dynamics and Self-Organization, Göttingen, Germany; 3 Key Laboratory of Evolution & Marine Biodiversity (Ministry of Education) and Institute of Evolution & Marine Biodiversity, Ocean University of China, Qingdao, China; 4 Department of Biology, Philipps University, Marburg, Germany; 5 The Gurdon Institute and Department of Genetics, University of Cambridge, Cambridge, UK; 6 Institute for the Dynamics of Complex Systems, University of Göttingen, Göttingen, Germany; 7 Max Planck Institute for Multidisciplinary Sciences, Göttingen, Germany; 8 Bernstein Center for Computational Neuroscience Göttingen, Göttingen, Germany

## Abstract

Cell junction remodeling is central to epithelial morphogenesis and tissue rheology and depends on the interplay between adhesion molecules and the actomyosin cortex. E-cadherin constitutes the molecular basis for epithelial cell adhesion, whereas cortical actomyosin plays a major role in intracellular force generation. However, the precise nanoscale organization and relationship between F-actin and E-cadherin at the cell interface still remain insufficiently understood. Here, we applied super-resolution DNA/peptide-PAINT microscopy to reveal the nanoscopic clustering of E-cadherin and its junctional distribution in relation to cortical F-actin at adherens junctions in the *Drosophila* embryonic epidermis. We were able to resolve distinct pairs of E-cadherin clusters approximately 45 nm apart on opposite sides of the adherens junctions. Intriguingly, these paired clusters were interspersed with unpaired clusters, lacking corresponding counterparts across the junction. We observed that cluster size, spatial arrangement, and cross-junction matching change during development and depend on N-glycosylation, a posttranslational modification affecting E-cadherin. Moreover, the organization of F-actin cortices between neighboring cells was found to be strongly correlated at junctions. Contrary to expectations, this intercellular F-actin correlation was observed independently of E-cadherin. Our study provides new insights into the nanoscale organization of adherens junctions, opening a window into the molecular mechanism of adhesion and mechanics of epithelial cells during morphogenesis.

## Significance

Cell-cell interfaces are fundamental to multicellular behavior, mediating adhesion and integrating biochemical and mechanical signals. Deciphering their molecular architecture is crucial for understanding epithelial morphogenesis, where coordinated cell behavior relies on precise junctional regulation. Here, we use DNA-PAINT super-resolution microscopy to resolve nanoscopic E-cadherin and F-actin organization at adherens junctions in *Drosophila* embryonic tissue. Being able to separately visualize both junction sides, we reveal that E-cadherin forms paired and unpaired nanoclusters, whose arrangement evolves during development and is modulated by N-glycosylation. Notably, we observe a degree of independence between F-actin and E-cadherin clustering, suggesting that intercellular cytoskeletal organization is not strictly dictated by only E-cadherin. These findings advance our understanding of junctional organization and its role in epithelial morphogenesis.

## Introduction

Epithelial cells are mechanically linked by adherens junctions, which were initially defined by electron microscopy as an interface of two cells due to a narrow gap between juxtaposed plasma membranes (1,2,3). The transmembrane protein E-cadherin is at the center of the molecular mechanism of adhesion by binding to an E-cadherin molecule from the adjacent cell, so-called *trans* homodimers (4,5,6). On the intracellular side, E-cadherin forms a tetrameric complex with p120-catenin, β-catenin, and α-catenin, which binds to F-actin and other actomyosin-associated proteins. Thus, a mechanical link is established between the contractile actomyosin cortices of two adhering cells (5,7,8).

In vivo*,* this core module comes in variations upon a theme depending on cell type and mechanics of the tissue. In addition to homodimers in *trans* complexes, up to hundreds of E-cadherin molecules can laterally associate within the same plasma membrane to form supramolecular *cis*-complexes with a characteristic size distribution (9,10,11). Such *cis* clusters are found at the lateral cortex in spot junctions or at the junction belt typical for many epithelial tissues. *Cis* clusters are also found at the free surface of the plasma membrane or in nonadherent cells without being engaged in trans homodimers and without an obvious function for adhesion (12,13,14). It is conceivable that such unpaired E-cadherin clusters are also present in adherens junctions. The fraction of E-cadherin molecules that are not engaged in *trans* complexes, and thus would not directly contribute to force transmission and adhesion, has remained unclear.

Adherens junctions are dynamic structures that assemble when two epithelial cells initiate contact or change their contractility (7). The obvious signature of junction formation and following maturation is an accumulation of E-cadherin complexes and associated cortical F-actin at the junctions (15,16). Similarly, contractility leads to increased levels of protein at the junction (17,18). It is assumed that initially clusters form and engage in localized *trans* complexes and then coalesce into larger structures that finally form a regular array at the junction belt. The dynamics of junction maturation has been difficult to directly assess given the size in the nanometer scale (19,20). Changes during maturation have remained unclear, for example, how unpaired *cis* clusters arrange along junctions; what fraction of clusters engage in *trans* complexes; and how E-cadherin clusters relate to cortical F-actin.

Early embryonic development provides an assay for stereotypic and physiological junction formation and maturation. In *Drosophila* embryos, adherens junctions initially form after about 3 h of development, during cellularization, and mature in the following hour during gastrulation, forming a typical junction belt (21,22). Genetic analysis of junctions is challenging and relies on partial loss-of-function mutations because no adherens junctions form in the epidermis when E-cadherin is lacking (2,23,24,25). We have previously observed that hypo-N-glycosylated E-cadherin impairs some aspects of E-cadherin function, whereas others, such as adhesion, appear normal (26).

Studies employing super-resolution microscopy have previously resolved clusters of E-cadherin and a hierarchy of junction-associated proteins (9,13,16,27,28). Imaging of adherens junctions is a great challenge in epithelial tissues in a physiological or developmental context since it requires a focal plane multiple micrometers deep into the tissue (29).

Here, we employ DNA/peptide-PAINT to investigate the nanostructure of E-cadherin clusters together with cortical F-actin during the maturation of adherens junctions in *Drosophila* embryos. DNA-PAINT is a technique based on single-molecule localization microscopy that leverages short-lived hybridization of DNA oligonucleotides to generate “blinks” for precise localization of single molecules. By attaching DNA “docking” strands to molecules of interest and inducing transient binding with DNA “imager”strands, a distinct fluorescence signal is generated.

Although DNA-PAINT has been established for flat samples (30), its application in thick tissues remains a challenge. One major limitation arises from the use of TIRF optics, which hinders imaging deep within tissue and thick samples. Two recent reviews have provided a comprehensive understanding of the imaging technique (31,32).

To address the issue of tissue imaging, we employed a spinning disk for optical sectioning, instead of the usual TIRF, allowing positioning of the imaging plane several micrometers deep into the tissue (33). In this work, E-cadherin and F-actin were imaged simultaneously using salvaged fluorescence microscopy (34) combined with DNA-Paint. In salvaged fluorescence microscopy, both probes are selected to spectrally overlap, and their emission is recorded and localized using the same, primary, detection channel while a secondary channel is used for color assignment only. In this way, both probes appear simultaneously in the same detection channel (same camera region of interest (ROI)) and are inherently registered. This eliminates the need for postprocessing channel registration. Any system or sample aberrations are equally shared by both probes as they both use the same detection channel and have closely matched emission spectra.

An intrinsic problem of DNA-PAINT is labeling efficiency, which has been estimated to be in the range of 20%–60% for nuclear pore proteins, depending on the labeling method (33,34,35). However, it is unclear whether the same labeling efficiency applies to target molecules in diverse molecular environments. Here, we assume a uniform labeling efficiency. In this way, we imaged E-cadherin and F-actin in the lateral epidermis during multiple stages of junction maturation in wild-type and mutant embryos with hypo-N-glycosylated E-cadherin. We quantified the apparently stochastic spatial distribution of E-cadherin and F-actin in relation to each other with mathematical measures for pattern similarity and correlation analysis.

## Materials and methods

### Genetics

Genetic markers and annotations are described in Flybase (36). Fly stocks were obtained from the Bloomington Drosophila Stock Center (37), if not otherwise noted. In the E-cadherin-GFP line, GFP sequence is inserted at the 3′ end of the coding sequence of the *shotgun* locus (38). Homozygous embryos were employed avoiding a competition of tagged and un-tagged E-cadherin molecules, also in *xit* embryos. *xit* encodes a glucosyl transferase of the ER catalyzing the second-to-last step in dolichol-N-glycan biosynthesis (39). *xit* mutants are characterized by hypo-N-glycosylated E-cadherin (26). *xit* embryos were derived from *ovo*^*D*^ selected germline clones (26) and were homozygous for E-cadherin-GFP. E-cad^NQ3x^ contained asparagine to glutamine mutations at three N-glycosylation sites, N466, N983, and N1274. E-cad^NQ4x^ contained asparagine to glutamine mutations at four N-glycosylation sites, N317, N949, N99, and N1290. An E-cadherin-GFP targeting vector was mutated and inserted into an attP site in the *shotgun* locus by phiC/attB-mediated transgenesis (40). The *white*^+^ selection marker was excised by Cre/loxP. A study with a systematic analysis of N-glycosylation sites in E-cadherin will be published elsewhere.

### Microscopy

Embryos were fixed by standard procedures and stained by GFP nanobody 453 (GFP-booster, ATTO488, ChromoTek, 1:200). Stage embryos were heat fixed in a salt solution (0.4% NaCl, 0.03% Triton X-100) and immunostained with an anti-Zipper antibody (rabbit, 1:2000) (41) for myosin II and an anti-GFP (chicken, 1:1000, abcam) for E-cadGFP. Embryos were imaged at a confocal microscope with Airyscan detector (Zeiss LSM980) and a 63×/1.4 oil objective. Images were deconvolved with ZEN software (Zeiss) (42).

The image stacks that covered the adherence junction plane were processed with the “max intensity” in ImageJ/Fiji. To quantify the intensity of junctional myosin II (in Fig. S2 *B*), the processed images were segmented using the “Tissue Analyzer” (43), a plugin of ImageJ/Fiji (44). Segmentation was used to restrict the ROI of the cell junctions, and the vertices (tricellular junctions) were excluded. The mean intensity from each junction and its angle with the AP axis were measured. The planar polarity (in Fig. S2 *C*) is the ratio between the intensity of each junction in the 60°–90° bin (angle with the AP axis, vertical cell border) and the mean intensity of the junctions in the 0°–30° bin (angle with the AP axis, horizontal cell borders) in each embryo.

### Nanoscopy

Embryos were fixed in a mixture of n-heptane and 8% formaldehyde (methanol free, Thermo Scientific) in phosphate-buffered saline (PBS) for 40 min, manually removed from the vitelline membrane, and stored in PBS at 4°C. For staining, embryos were permeabilized with 0.1% Triton X-100 in PBS for 1 h at 4°C, blocked with 5% bovine serum albumin in PBS for 30 min at room temperature, and incubated with 20 nM GFP nanobody conjugated with an oligonucleotide (docking strand, FluoTagQ anti-GFP, clone 1H1, docking site 3, Massive Photonics) in PBT (PBS with 0.1% Tween-20) for 2 h at room temperature. After washing with PBT over 1 h at room temperature, embryos were mounted on chambered glass cover slides (IBIDI) treated with Cell-Tak (Corning). The imaging focal plane was set using the GFP fluorescence from E-cadherin-GFP. For recording, the chambers were filled with imaging solution (Massive Photonics) containing 1 nM imager oligonucleotide, which was complementary to the nanobody docking strand and labeled with the fluorescent dye Cy3B.

For two-color recordings, the imager oligonucleotide for GFP was conjugated with Atto594 while the peptide Lifeact was conjugated with Cy3B. Concentrations between 1 and 5 nM were used for both probes. 60k unprocessed camera frames were collected in blocks (cycles) of 1000 frames at a frame rate of 5 Hz on an inverted microscope (Olympus IX83) with a spinning disk with a 100-μm spiral pattern (Custom, CrestOptics) and a 100× objective (NA 1.35, silicone oil immersion, Olympus, UPLSAPO100XS). Blink detection, real-time and postprocessing drift correction, and image reconstruction were conducted as described previously (33). For dual-channel recordings, blinks were assigned to the F-actin or E-cadherin channel using salvaged fluorescence (34). In salvaged fluorescence microscopy, both probes are selected to spectrally overlap, and their emission is recorded and localized using the same, primary, detection channel while a secondary channel is used for color assignment only. In this way, both probes appear simultaneously in the same detection channel (same camera ROI) and are inherently registered. This eliminates the need for postprocessing channel registration. Any system or sample aberrations are equally shared by both probes as they both use the same detection channel and have closely match emission spectra. Typical localization precision was <20 nm, and super-resolved images were reconstructed by creating a 2D histogram using the blink (x, y) positions resulting in final images with a 10-nm pixel size.

The validity of salvaged fluorescence imaging was experimentally verified using *Drosophila* egg chambers with two tagged proteins in the nuclear pore complex (NPC) as previously show in (33). NPCs are a well characterized structure used by many super-resolution microscopists as a standard target to evaluate microscope and postprocessing performance. Specifically, we imaged Nup160-SNAP and Gle1-Halo on the nurse cell nuclei and through the epithelia cell layer. Nup160 labels the NPC ring while Gle1 marks the center. In our imaging experiments, Gle1 appears at the center of the Nup160 rings across a 20 × 20 μm^2^ field of view.

### Software

For raw data processing, we used Fiji/ImageJ (44). Otherwise, analysis was conducted with custom written software in Python 3.10.

### Raw data processing

The original raw data were manually segmented and processed using Fiji. The midpoint of each junction was traced using the segmented line tool. An ROI was defined by an area with 31 pixels width around the created backbone. Using the “straighten” tool, the junction is straightened (Figs. S3 *A* and S8). To evaluate the impact of the straighten operation on the original signal, we chose a sample cluster (Fig. S8 *B*) and drew segmented lines of different curvature that were subsequently straightened. We calculated the local curvature from the segmented line and the width of a sample cluster by fitting a Gaussian to the intensity signal (Fig. S8 *A*). Then, we compared the width before and after straightening (Fig. S8 *C*). We further calculated local curvature along original segmented junctions and plotted the distribution (Fig. S8 *D*). For all genotypes, the mean curvature of junctions is only subject to negligible deformation, showing that the majority of local junction segments are linear. The strongest curvatures are found in *xit* mutants, where most clusters are still well below 25% total deformation.

### Thresholding

Some of our quantifications require thresholding of the DNA-PAINT-generated intensities. We determined a threshold using intensity values 15 pixels (150 nm) away from the junction center on both sides, which serves as a proxy for the background signal. From the distribution of those intensities, we calculated the 95% bootstrapped confidence interval. Every intensity value above the upper confidence level of 97.5% is assumed to be significantly different from the background and is included in the analysis. All other pixels with values below the significance threshold are not included.

### Spatial profile

The average intensity orthogonal to the membranes was calculated per 1000-nm patch along the junctions. Across all patches, we bootstrapped the average intensity profile to obtain error regions. The average intensity is fit with two Gaussians. The distance between the two membranes is estimated by the distance between the two maxima.

### Correlation analysis

Straightened junctions were divided in two regions “left” and “right” from the center. The center pixel was not included in the analysis. We again performed analysis in 1000-nm patches along the junction. Intensity values were approximately exponentially distributed. Hence, we took the logarithm of the intensity signal before the correlation analysis. This makes sure uncorrelated signals go to zero, and the correlation assumes values between −1 and +1 as usual. For all pixels up to 50-nm width, which corresponds to the half-point of the intensity profiles (Fig. 3 *A*), we summed the intensity values to obtain a one-dimensional representation of the E-cadherin intensity for each side of the junction. The correlation function is obtained by calculating the cross-covariance function (45) and normalizing with the standard deviations of each respective signal. Correlation coefficients correspond to the normalized covariance function at lag = 0 nm. The covariance/correlation functions for each patch are averaged per mutant. The error region is obtained by computing the 95% confidence intervals using bias-corrected and accelerated bootstrap.

### Expected correlations of local coupling model

We wanted to know the expected cross correlation of F-actin signals on both junction sides assuming that they can only be connected via local links with E-cadherin. In a simple Markov model the expected correlation over several links can be calculated from the measured correlations of directly linked nodes in the chain. Assuming Gaussian noise *x* with zero mean and standard deviation equal to one, the intensity signals are given by

$$\left(\begin{array}{c} a_{1}\\ c_{1}\\ c_{2}\\ a_{2} \end{array}\right)=\sqrt{Ĉ}\left(\begin{array}{c} x_{a1}\\ x_{c1}\\ x_{c2}\\ x_{a2} \end{array}\right)$$

with the correlation matrix

$$Ĉ=\left(\begin{array}{cccc} c^{a1a1} & c^{a1c1} & c^{a1c2} & c^{a1a2}\\ c^{c1a1} & c^{c1c1} & c^{c1c2} & c^{c1a2}\\ c^{c2a2} & c^{c2c1} & c^{c2c2} & c^{c2a2}\\ c^{a2a1} & c^{a2c1} & c^{a2c2} & c^{a2a2} \end{array}\right)$$

In the local model, we know the direct correlations a_1_-c_1_, c_1_-c_2_, and c_2_-a_2_. The nonlocal correlations can then be calculated by

$$c^{c_{2}a_{1}}=c^{c_{1}a_{1}}\cdot \;c^{c_{2}c_{1}}$$

$$c^{c_{1}a_{2}}=c^{c_{2}c_{1}}\cdot \;c^{a_{2}c_{2}}$$

$$c^{a_{1}a_{2}}=c^{c_{1}a_{1}}\cdot \;c^{c_{2}c_{1}}\cdot \;c^{a_{2}c_{2}}$$

### Overlap

Using the same data as for the correlation analysis, we computed the fraction of coupled E-cadherin by the overlap in the intensities from both sides of the junction. To this end, we binarized the signal by significance thresholding. Since there is no unique way of obtaining an overlap, we compared several methods. We call the sets of significant pixels from both sides X and Y. Their length, i.e., the number of pixels, is denoted by |X| and |Y|. Then, Mander M1 and M2 coefficients are defined by

$$M1=\frac{\left|X\cap Y\right|}{\left|X\right|},M2=\frac{\left|X\cap Y\right|}{\left|Y\right|}$$

The “symmetrized” version of Mander coefficients is the Dice coefficient (Fig. 3 *E*).

$$DC=\frac{2\left|X\cap Y\right|}{\left|X\right|+\left|Y\right|}$$

Finally, a third possibility to quantify the overlap is the intersection over union (IoU), which is defined by

$$IoU=\frac{\left|X\cap Y\right|}{\left|X\cup Y\right|}$$

### Cosine similarity

To compare the two-dimensional pattern between both sides of the junction, we first divided the junction into two 1000-nm-long and 50-nm-wide patches. One of the patches is mirrored along the 50-nm axis to make them comparable to each other. The intensity values in the patch are then flattened into a vector that has the dimension of the number of pixels in the patch. A similarity of two vectors can be given in terms of the angle between them. Two vectors are more similar if they point in the same direction. This is formalized by the cosine similarity (Fig. 3 *F*), which is the cosine of the angle between the vectors and is defined as

$$\cos\left(\alpha \right)=\frac{v_{1}v_{2}}{\left|v_{1}\right|\left|v_{2}\right|}\text{,}$$

with *v*_1_ and*v*_2_ being the vectors containing the intensities. The cosine similarity yields a number between −1 and 1.

### Patch comparison method

To assess differences in spatial arrangements between the difference genotypes and developmental stages, we utilized the comparison of randomly drawn 1000-nm-long patches from junction segments. As described above, patches can be represented by intensity vectors by flattening the array of each patch (Fig. 4 *E*). The structural differences between two patches are calculated using cosine similarity as described above. In addition, we evaluated the differences in patch intensity using the length of the intensity vectors. To obtain globally valid comparison of the vector length differences, we first obtained the length of each vector using the usual vector norm and then calculated differences in length and finally normalized the result using a sigmoid function.

$$S\left(x\right)=\frac{1-e^{-x/\lambda }}{1+e^{-x/\lambda }}$$

This normalization bounds the length differences between −1 and 1. We empirically chose the same scaling of the sigmoid for all evaluations.

Given the distance of two local patches, the overall similarity of two patch ensembles was calculated using Wasserstein distance.

$$W\left(\mu_{0},\mu_{1}\right)=\min_{T\in R^{k_{0}\times k_{1}}}\;\sum_{\text{ij}}\;T_{\text{ij}}\;\left|x_{0i}-x_{1j}\right|\text{,}$$

where x denotes patches with numbers i and j from the two patch ensembles μ_0_ and μ_1_, and *T*_*ij*_ is the optimal transport matrix. *T*_*ij*_ is optimized such that the sum of all its entries times the patch distance is minimal (Fig. S5 *F*). This number is then called the Wasserstein distance. It is bounded from below by 0, which indicates coinciding distributions. The more dissimilar two spatial arrangements are, the larger the value of the Wasserstein distance will become.

### Entropy

The (discrete) Shannon entropy *S* = ∑*p*_*i*_*log*_2_*p*_*i*_, where *p*_*i*_ are normalized frequencies, serves as a measure of uncertainty in information theory and statistical physics (46,47). It depends on the shape of a probability distribution where unimodal sharp distributions yield lower values, and uniform distributions, or distributions with many peaks, yield high entropy values. We calculated the Shannon entropy of each 1000 nm × 100 nm patch along junctions by treating the E-cadherin intensity signal as a two-dimensional probability distribution. Intensities were normalized to obtain a “probability” of the E-cadherin locations $p_{i}=I_{i}/\sum_{i}I_{i}$ where the index *i* runs over all pixels in the patch. Because the entropy is extensive, meaning it depends on the system size, quantifications are only comparable when using the same patch size with the same number of pixels. Our pixel size is 10 nm × 10 nm.

### Cluster segmentation

We identified local E-cadherin clusters by assuming that each E-cadherin cluster gives rise to a unique maximum in the DNA-PAINT-generated intensities. We assigned groups of pixels to each maximum (cluster) by use of a watershed algorithm. To this end, we first detected local maxima of the E-cadherin signal using a maximum filter. Before applying the maximum filter, the image was slightly smoothed to avoid detection of insignificant local extrema, using a Gaussian filter with sigma = 5 nm width. The resulting maxima were used as seeds for a watershed algorithm, implemented in scikit-image (48), which performs the segmentation of the image into pixel groups corresponding to one cluster. Pixels with an intensity value below the significance threshold (see above) were excluded from the analysis.

### Cluster analysis

We obtained a measure for the number of E-cadherin molecules per cluster by summing all intensity values in each segmented pixel group. Intensities are directly related to the number of E-cadherin molecules, save a scaling factor, which depends on the calibration of the experimental setup and is constant across all trials. See also the discussion in the main text. We inferred parameters for the cluster size distribution by assuming a power law with exponential cutoff.

$$\rho \left(n\right)=A\;n^{\alpha }\;e^{-n/n^{*}}$$

*n* is the cluster size, here the sum of intensities in a pixel group, α is the scaling of the power law, and *n*^∗^ determines the exponential cutoff. Such a distribution has been previously analyzed in the context of experimental E-cadherin size distribution and also derived from a clustering model (9). This functional form appears generically for clustering models in many circumstances, for example for size distributions of postsynaptic scaffold domains (49,50,51,52). In addition, we performed a model comparison to test the power law hypothesis against a simple exponential distribution.

For parameter inference, we used the Bayesian inference scheme nested sampling, as implemented for Python in the module “dynesty” (51,52). Nested sampling can directly sample from the posterior and evidence and can deal with complex parameter dependencies, which we expect due to the natural coupling between α and *n*^∗^ in the power law with exponential cutoff. We generated a histogram of the cluster size distribution using logarithmic binning and sample from a least squares likelihood between the empirical histogram and the respective model probability distribution.

### Statistical tools

If not stated otherwise, error regions and significances were derived from 95% confidence intervals, which were calculated using bias-corrected and accelerated bootstrap.

If not stated otherwise, parameter inference and model comparison has been performed using the Bayesian inference technique nested sampling. Bayesian statistics utilizes Bayes law to estimate the probability of a parameter given an observation (data), which is called the posterior in Bayesian jargon.

$$P\left(\Theta |D,M\right)=\frac{P\left(D|\Theta ,M\right)P\left(\Theta |M\right)}{P\left(D|M\right)}$$

Hereby, Θ are the model parameters, *D* is the data, and *M* the model. To obtain the posterior, one has to input a prior probability *P*(Θ|*M*), which we choose to be uniform, and a likelihood L=P(D|Θ,M) where the comparison between data and model enters. If not indicated otherwise, we used a simple Gaussian likelihood:

$$\log\;L=-\frac{1}{2}\;\log\;\left(2\;\pi \;\sigma^{2}\right)-\frac{1}{2}\;\sum_{i=1}^{N}\;{\left(y_{\text{data}}^{i}-\mu^{i}\right)}^{2}\text{,}$$

where μ(Θ) is obtained from the model. Parameters are estimated as the median (50 percentile) of the estimated posterior distribution. To obtain estimation errors, we computed 95% credible intervals by taking the 2.5th and the 97.5th percentile of the posterior distribution as lower and upper bound for the estimated parameter.

For model comparison, we used the evidence $Z_{i}=P\left(D|M_{i}\right)\text{,}$ which is simultaneously estimated by nested sampling during posterior estimation. The Bayes factor $B=\frac{Z_{1}}{Z_{2}}$ is a commonly used metric that essentially gives an estimate of how much more likely model 1 is in comparison to model 2, given the same observation (data).

### Fluorescence recovery after photobleaching

Embryos were prepared as previously described (53). Staged embryos were collected and dechorionated with 50% hypochlorite bleach for 90 s, aligned on an agar block, and attached to the coverslips by homemade glue covered with halocarbon oil. A cross section with a frame rate of 1 Hz was acquired using laser scanning confocal microscopy (ZEISS LSM 980 with Airyscan 2, Carl Zeiss) with a 63× oil objective (63×/oil, NA1.4, Carl Zeiss). The focal plane was set to 1 μm. The image size is 33.67 × 33.67 μm (512 × 512 pixel). The bleaching ROI was for E-cadherin clusters with 0.5 × 1 μm. The ROIs were bleached by 50% of 488 laser intensity with 20 iterations after recording two frames. The ROI was tracked to quantify E-cadherin-GFP recovery after photobleaching, and the fluorescence intensity of ROIs was measured manually in Fiji/Image J. The fluorescence intensity was normalized in each recording by

$$I=\frac{\left(It-Imin\right)}{\left(Imax-Imin\right)}\text{,}$$

where I_t_ represents the intensity at time t; I_min_ represents the intensity of the first frame after bleaching; I_max_ represents the intensity of one frame before bleaching.

The mobile fraction and half-time were calculated by “one-phase association” fitting from normalized fluorescence intensity after bleaching in GraphPad Prism 10 (54).

Y=Y0+(Plateau−Y0)∗(1−exp(−K∗x)),

where

Y0=0andK>0.

### Laser ablation

Embryos were prepared as previously described (53). Staged embryos were collected and dechorionated with 50% hypochlorite bleach for 90 s, aligned on an agar block, attached to the coverslips by homemade glue, with desiccation for 5 min, and covered with halocarbon oil. The E-cadGFP channel was recorded with 1 Hz by using laser scanning confocal microscopy (ZEISS LSM 980 with Airyscan 2, Carl Zeiss) with a 63× oil objective (63×/oil, NA1.4, Carl Zeiss). A 355-nm pulsed laser (DPSL355/14, 355 nm, 70 μJ/pulse, Rapp OptoElectronic, Hamburg, Germany) was employed for ablation and manipulated on the “REO-SysCon-Zen” platform (Rapp OptoElectronic). The 355-nm pulsed laser was mounted on the epiport of an LSM 980. Laser ablation was performed with 5% laser power, with a 100-ms (approximately 20 pulses) exposure time at the vertical cell contacts. The distances between the tricellular junctions flanking the ablated contact were measured manually as the “displacement length” in Fiji. The displacement values were normalized to the initial length [L(0)] and plotted over time. The initial recoil velocities (in Fig. S2 *F*) and viscoelasticity (in Fig. S2 *G*) were calculated by fitting as a Kelvin-Voigt fiber model (55) to the following equations in GraphPad Prism10 (56).

Extraction of initial recoil values:

$$\varepsilon \left(t\right)=L\left(t\right)-L\left(0\right)=\frac{F0}{E}\;.\;\left(1-e^{-\left[\left(\frac{E}{\mu }\right)*t\right]}\right)$$

Here, F_0_ is the tensile force present at the junction before ablation, E is the elasticity of the junction, and μ is the viscosity coefficient related to the viscous drag of the cell cytoplasm.

The fitting parameters for the above equation were as follows:

$$initial\;recoil=\frac{d\varepsilon \left(0\right)}{dt}=\frac{F0}{\mu }$$

and

$$K=\frac{E}{\mu }$$

## Results

### Imaging by DNA/peptide-PAINT reveals transmembrane spatial organization of E-cadherin and F-actin

**Figure 1:**
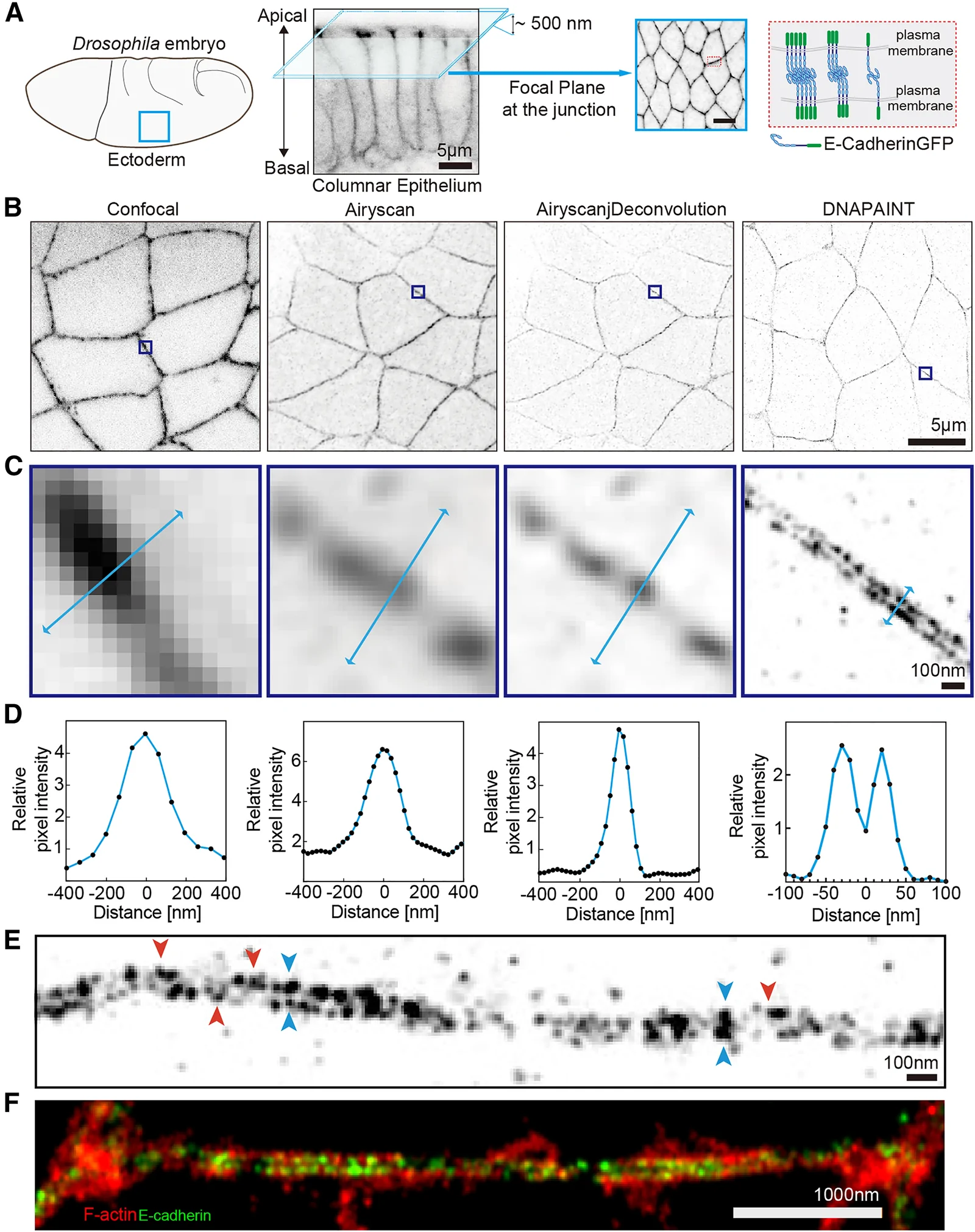
Transmembrane spatial organization of E-cadherin is revealed by DNA/peptide-PAINT. (*A*) Imaging in the germband of *Drosophila*. We have a 20 × 20 μm field of view with roughly 500-nm axial resolution to resolve junctional E-cadherin. (*B*) Representative images comparing confocal, Airyscan, Airyscan jDeconvolution, and DNA/peptide-PAINT microscopy. Scale bar represents 5 μm. (*C*) Magnified junctions as indicated by blue square in (*B*) for all four recording techniques. Scale bar represents 100 nm. (*D*) Intensity profiles across the junctions corresponding to the blue lines in (*C*). The intensity has arbitrary units and cannot be compared across different methods. (*E*) Magnified image from DNA/peptide-PAINT recording. Blue arrows indicate paired E-cadherin complexes across the membrane. Red arrows denote unpaired E-cadherin sites. This classification is only visual and is generalized using a cross correlation framework in the following work. Scale bar represents 100 nm. (*F*) Image of simultaneously recorded F-actin and E-cadherin at a junction. Scale bar represents 1000 nm.

We used the single-molecule localization microscopy technique, DNA/peptide-PAINT, to image E-cadherin and F-actin in gastrulating *Drosophila* embryos (30,33) (Fig. 1 *A*). Our focus was on developmental stages 6–8 when the adherens junctions are maturing and epithelial cell contacts are actively remodeling. Specifically, we used a GFP nanobody conjugated to an oligonucleotide and the complementary imager strand oligonucleotide fused to a fluorescent dye to visualize endogenously tagged E-cadherin-GFP (38). Our microscopy setup and computational pipeline achieved localization precisions of less than 15 nm within the focal plane (33). Confocal optics is achieved with a spinning disk allowing axial positioning several micrometers into the tissue with an axial resolution in the range of ∼500 nm (33,57).

We imaged adherens junctions in the lateral epidermis, where the spatial/directional information of the junctions was not collected during imaging, by both conventional and high-resolution confocal microscopy and compared the results to those obtained using DNA/peptide-PAINT (Fig. 1 *B*–*D*). Confocal microscopy revealed a single line of lateral clusters of various sizes greater than ∼100 nm. In contrast, DNA-PAINT resolved both juxtaposed sides of the junction (Fig. 1 *B*–*D*). We detected two traces along the junction consisting of E-cadherin clusters of varying intensities, which align with the borders of the two adhering cells. Representative line profiles perpendicular to the junction illustrate the improved resolution (Fig. 1 *D*). Although the three types of confocal microscopy produced a single maximum, two peaks were resolved in the DNA/peptide-PAINT images. The two peaks indicate a distance of approximately 50 nm between the distal ends of *trans* complexes, consistent with an intermembrane distance of ∼30 nm from EM images (2,3,58) (Fig. 1 *C* and *D*).

Visual inspection revealed an unexpected distribution of the E-cadherin clusters along the junctions in the embryonic epidermis. Paired clusters, in which an E-cadherin cluster in one membrane directly abutted a cluster in the other membrane, were dispersed throughout unpaired E-cadherin clusters (Fig. 1 *E*). With an axial depth of the images of ∼500 nm, tilted *trans* complexes will appear as paired clusters. Thus, the fraction of observed unpaired clusters provides an underestimate of the actual number of clusters without a corresponding partner in the other cell. Despite the relatively thick optical sections, clusters were clearly resolved. This suggests that E-cadherin coalesces into a rather narrow stripe at the transition from the subapical to lateral cortical domain.

Besides E-cadherin, cortical F-actin constitutes a major component of adherens junctions. We imaged F-actin with a fluorescently labeled Lifeact peptide, which has binding kinetics suitable for single-molecule localization microscopy (33,59). We recorded E-cadherin and Lifeact simultaneously and registration free by spectral separation of each blink after excitation with a single laser line. Similar to E-cadherin, F-actin was detected clearly separated for the two sides of the junction (Fig. 1 *F*). The spatial organization of both molecules featured an apparent degree of randomness. In particular, the relationship between F-actin and E-cadherin was difficult to assess visually and showed no obvious correlation (Fig. 1 *F*).

Based on our initial, qualitative assessment of E-cadherin and F-actin distribution, we hypothesized that paired E-cadherin clusters with presumably many homotypic *trans* complexes are dispersed among unpaired E-cadherin clusters in a stochastic pattern at adherens junctions. Based on previous functional and microscopic data, we expect to find that E-cadherin clusters and F-actin are correlated (7,13). We also expect that the stochastic pattern changes with junction maturation. Furthermore, partial loss-of-function of E-cadherin molecules by hypo-N-glycosylation may impinge on the pattern and pairing of clusters and its relation to cortical F-actin.

Before testing these hypotheses, we briefly describe the N-glycosylation mutants that serve as comparison to wild-type embryos.

### Hypo-N-glycosylated E-cadherin impairs mobility and mechanical properties of junctions

We chose to include several N-glycosylation mutants into our study since previous experiments revealed a function of N-glycosylation for E-cadherin and tissue dynamics during gastrulation. Mutants of the N-glycan biosynthesis pathway, such as *xit*, lead to hypo-N-glycosylation of E-cadherin and potentially many other protein passing through the ER and impaired cell intercalation (26,60). To test the role of N-glycosylation for E-cadherin, we systematically mutated the N-glycosylation sites of E-cadherin at its endogenous locus (NQ alleles, Asn to Gln) (8,61). Here, we analyzed two E-cad^NQ^ alleles with three or four NQ mutations at different sites (Fig. S1 *A*). A systematic description and functional characterization of E-cadherin N-glycosylation mutants will be reported in a separate study.

As previous studies showed, the mobile fraction of E-cadherin as measured by fluorescence recovery after photobleaching (FRAP) decreases from about 50% at stage 6 (onset of gastrulation) to 15%–20% half an hour later in stage 8, what may be due to an incorporation of molecules into stable clusters (62). We performed FRAP experiments in both E-cad^NQ^ alleles and *xit* mutants at stage 8 and found a large mobile fraction of 40%–50% in comparison to wild-type embryos with 24.9% (Fig. S1 *B*–*E*).

We detected reduced myosin II levels by Zipper antibody staining at junctions in both E-cad^NQ^ alleles and *xit* compared with wild-type, whereas the distribution pattern of plane polarity remained consistent. (Fig. S2 *A*–*C*). However, when we performed micro-dissection of junctions, we observed a higher initial recoil velocity in all three mutants compared with wild-type. There is no significant difference in the *K* values, which represent the ratio between junctional elasticity and medium viscosity; thereby the higher initial recoil velocity in the mutant embryos suggests higher tension (Fig. S2 *D*–*G*).

These experiments reveal that full N-glycosylation of E-cadherin contributes to its molecular behavior and the mechanical properties of adherens junctions. As they don’t obviously affect epithelium formation and cell adhesiveness the E-cad^NQ^ and *xit* mutants are suited for genetic variation of E-cadherin structure and function at junctions. The E-cad^NQ3x^ allele was included in the analysis with one channel, the E-cad^NQ4x^ allele, in the data set with E-cadherin and F-actin double labeling.

### Wider distance between distal ends of E-cadherin in mutant embryos

We imaged embryos during gastrulation, when the adherens junctions mature, and compared wild-type junctions to mutants with hypo-N-glycosylated E-cadherin. For comparison of various experimental situations, we quantitatively analyzed those images for various features concerning the arrangement of E-cadherin clusters. We generated two data sets: 1) single-channel E-cadherin-GFP for wild-type, *xit* and E-cad^NQ3x^ and 2) dual-channel E-cadherin-GFP and F-actin, for wild-type, *xit* and E-cad^NQ4x^. The data sets were analyzed separately because they were based on distinct imaging conditions.

**Figure 2:**
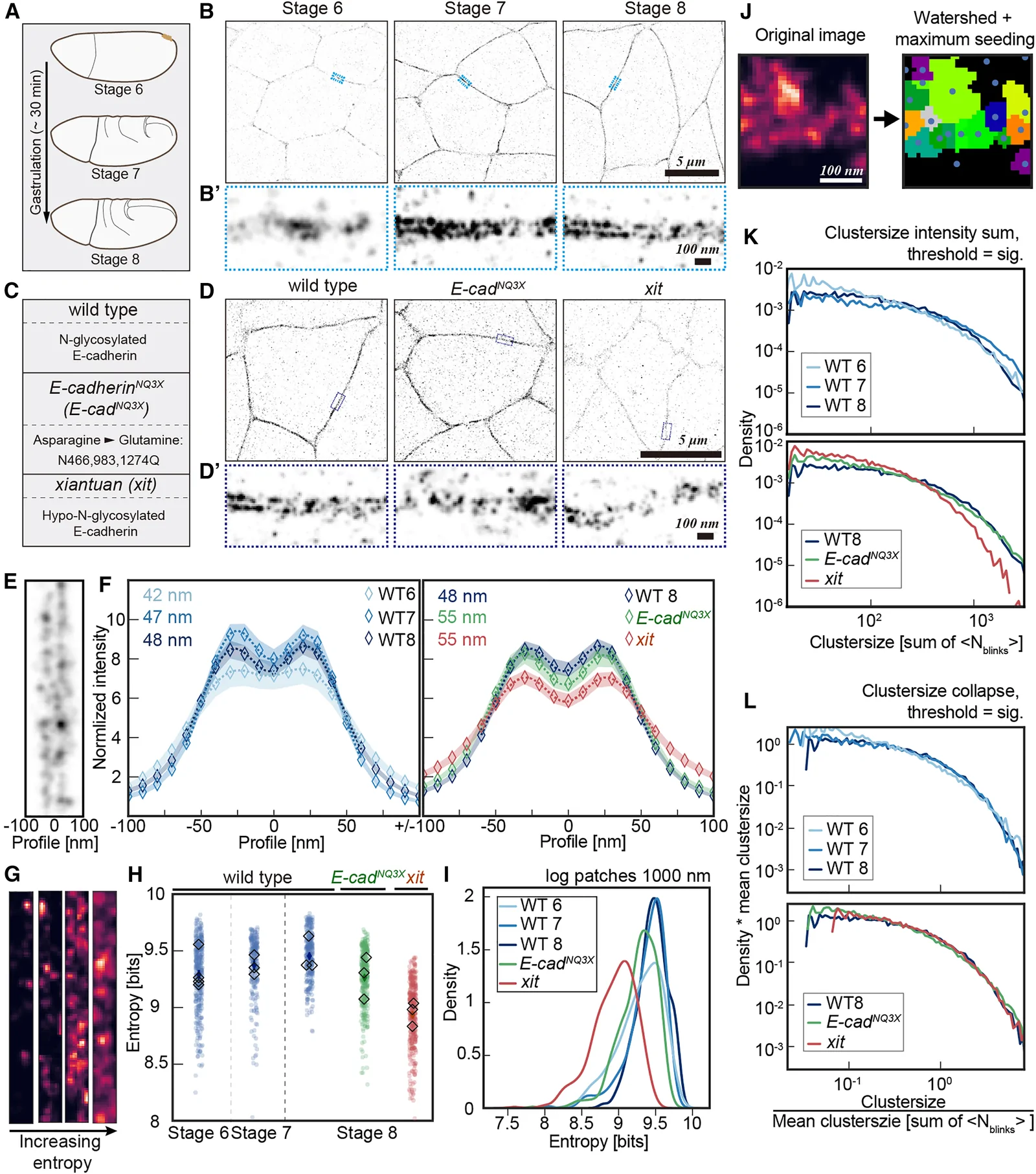
Quantification of E-cadherin clusters at adherens junctions during development and in mutants with hypo-N-glycosylated E-cadherin. (*A*) Depiction of gastrulation in *Drosophila* and the analyzed stages. (*B*) DNA/peptide-PAINT images of E-cadherin from the indicated stage embryos. Scale bars represent 5 μm. (*B′*) Highlighted junctions corresponding to blue rectangles in (*B*). Scale bar represents 100 nm. (*C*) Indication of mutation sites. (*D*) Representative images of wild-type, *E-cad*^*NQ3x*^, and *xit*. Scale bar represents 5 μm. (*D′*) Highlighted junctions corresponding to blue rectangles in (*B*). Scale bar represents 100 nm. (*E*) Example of a straightened junction. The midpoint between both membrane sides was manually segmented and serves as reference point of 0 nm of the profile. (*F*) Averaged normalized E-cadherin intensity as a function of the profile location displayed as diamonds. The shaded region is the 95% bootstrap confidence interval computed by subsampling average profiles from 1-μm-long segments. The number of junction segments are WT-stage 6 (four embryos): 370; WT-stage 7 (three embryos): 242; WT-stage 8 (three embryos): 324; *E-cad*^*NQ3x*^ (three embryos): 365; and *xit* (three embryos): 367. Dashed lines indicate maximum likelihood fits of two Gaussians to the profile. The estimated width of the membrane corresponding to the distance of the two fitted maxima is given. (*G*) Depiction of entropy as generalized measure of variability. Distributions with high entropy show broader peaks and high density of E-cadherin loci per segment, whereas for low entropy values the E-cadherin distribution is sparser and more localized. (*H* and *I*) Distribution of entropy values. Each point corresponds to a 1-μm-long junction segment with 100-nm width (same numbers as in *F*). The diamond is the average entropy value, and the bar indicates its 95% bootstrap confidence interval, where each box represents the average value from a single embryo. With the exception of WT6 and E-cad^NQ3x^, all distributions are significantly different from each other; also compare Fig. S7 and Table S1. (*J*) Scheme illustrating the watershed clustering method. Single E-cadherin loci (*clusters*) are identified by a maximum filter (*blue dots*). The watershed algorithm was seeded by the maxima, and we segmented the image according to the negative E-cadherin intensity. Pixels with intensities below a significance threshold were excluded. (*K*) Distributions of cluster sizes with logarithmic binning. The cluster size equals the integrated intensity in a segmented pixel group. The number of clusters are WT-stage 6 (four embryos): 9492; WT-stage 7 (three embryos): 10552; WT-stage 8 (three embryos): 11255; *E-cad*^*NQ3x*^ (three embryos): 17518; and *xit* (three embryos): 11927. (*L*) Distributions of cluster sizes normalized to mean cluster size.

We first analyzed the developmental changes in the nanoscale arrangement of E-cadherin clusters between stage 6 (onset of gastrulation), stage 7 (ongoing cell intercalation), and stage 8 (germ band extension) covering about half an hour of development (Fig. 2 *A*–*B′*) (21). Secondly, for stage 8, we compared wild-type to mutant embryos with hypo-N-glycosylated E-cadherin: embryos from *xit* germline clones and E-cad^NQ3x^ mutants (Fig. 2 *C*–*D′*).

We manually segmented cell junctions, traced the center of the junction, and computationally straightened the traces (Fig. S3 *A*). Our data set consists of about a hundred junctions with a total length of a few hundred micrometers from at least three embryos for each stage or genotype (stage 6, four embryos: 134 junctions, 437 μm; stage 7, three embryos: 77 junctions, 280 μm; stage 8, three embryos: 76 junctions, 362 μm; E-cad^NQ3x^, three embryos: 94 junctions, 411 μm; *xit*, three embryos: 90 junctions, 425 μm).

We averaged the intensities along the straightened traces (Fig. 2 *E* and *F*). The intensity profile perpendicular to the traces resulted in two peaks consistent with the profile in Fig. 1. To calculate the distance between the two peaks, we fitted two Gaussians to the profile and measured the distance between their maxima to obtain an averaged distance with 95% confidence intervals (Fig. 2 *F*): stage 6: 42 nm, CI_95_ = [37.7, 45.6] nm; stage 7: 47 nm, CI_95_ = [45.6, 48.2] nm; stage 8: 48 nm, CI_95_ = [46.8, 49.8] nm. For the mutants, we estimated a distance between peaks of the profile of about 55 nm, CI_95_ = [53.3, 55.9] nm in E-cad^NQ3x^ and 55 nm, CI_95_ = [52.7, 60.4] nm in *xit* embryos (Fig. 2 *F*). To further ascertain the detected differences, we obtained a Bayesian estimate of the two Gaussians for each peak of the profile using nested sampling. Credible intervals of the inferred mean confirmed that an increased distance for E-cad^NQ3x^ is supported by our data but not for *xit,* since the credible interval overlaps with the wild-type distances (Fig. S3 *B*).

A distance in the range of ∼45 nm between the distal ends of E-cadherin on both sides of a junction is consistent with electron microscopy data, which gives the distance between the two plasma membranes as ∼30 nm as well as molecular data for the distance between the distal ends of an E-cadherin *trans* complex (63). The slightly increasing distance from stage 6 to stage 8 and the ∼10 nm larger distance in the mutants indicates a diverse cortical structure. It is conceivable, for example, that some unpaired clusters are drawn toward the cortex, and thus the averaged profile of all paired and unpaired clusters would move further apart. We would not expect a change in the molecular architecture of the E-cadherin *trans* complexes.

### Entropy reveals changes in cluster arrangement in *xit* and E-cad^NQ3x^ mutants

We sought a simple metric to compare the complex pattern of E-cadherin clusters along junctions. Entropy generalizes the notion of variance of a distribution to arbitrary multidimensional and multimodal distributions. Hence, larger entropy values correspond to higher variability in the E-cadherin distribution and higher uncertainty about the location of single E-cadherin clusters (Fig. 2 *G*). Importantly, entropy is not sensitive to the total intensity level but a readout for the general shape of a distribution. We calculated the entropy of the intensity patterns in segments of 1 μm to obtain a single metric representative for cluster arrangement (Fig.2 *H* and *I*). We found that entropy slightly increased from stage 6 to stage 8 junctions (Figs. 2 *H* and *I*, and S7A; Table S1). In *xit* and E-cad^NQ3x^ mutants, we found many segments with reduced entropy compared with wild-type stage 8 (Figs. 2 *H* and *I*, and S7A; Table S1). Junction segments in later stages featured more evenly distributed E-cadherin organization along the junctions. In contrast, a broader distribution in stage 6 and the mutants could be explained by unevenly matured junctions where some segments already show stage 8 type organization, whereas other segments are still sparsely populated. This interpretation also becomes visually plausible when comparing the E-cadherin organization of the different stages (Fig. 2 *B*). Although entropy can be a simple metric to distinguish differences between complex patterns, we turn to a more detailed statistical analysis of the cluster sizes, which provides information about the underlying processes that govern E-cadherin association and dissociation at adherens junctions.

### Stage-dependent cluster size distribution suggests developmental regulation of E-cadherin dynamics

We identified clusters by segmentation of pixel groups in the recorded images and integrated intensities within the cluster area as a relative indicator of the number of molecules per cluster (Fig. 2 *J*). We found cluster sizes distributed according to a power law with an exponential cutoff $\rho \left(n\right)=A\;n^{\alpha }\;e^{-n/n^{*}}$ (Fig. S3 *C* and *D*). The distributions in wild-type stage 6 and in both *xit* and E-cad^NQ3x^ mutants were skewed toward smaller cluster sizes (Fig. 2 *K*).

Intriguingly, the distribution functions of mutants and wild-type collapsed to a single curve when normalized to the mean cluster size (Fig. 2 *L*). Thus, cluster size distributions of wild-type and mutants mainly differ by scaling to their respective average cluster size and generally follow the same functional law.

We found that in wild-type stages 7 and 8 the distribution is closer to an exponential distribution compared with stage 6 and both mutants. To further test the relation of exponential versus power law, we applied Bayesian model comparison and obtained the evidence for both the power law model with exponential cutoff and the pure exponential distribution (Fig. S3 *E*). The difference in evidence between the pure exponential distribution and the power law with cutoff is smaller in stages 7 and 8 than in stage 6 or mutants. The shift toward an exponential distribution indicates a developmental change in E-cadherin clustering. Therefore, nanoscopic methods may be key to understanding the kinematics that guide E-cadherin assembly and disassembly at adherens junctions and their regulation during development.

### Reduced pairing of E-cadherin clusters in *xit* and E-cad^NQ3x^ mutants

**Figure 3:**
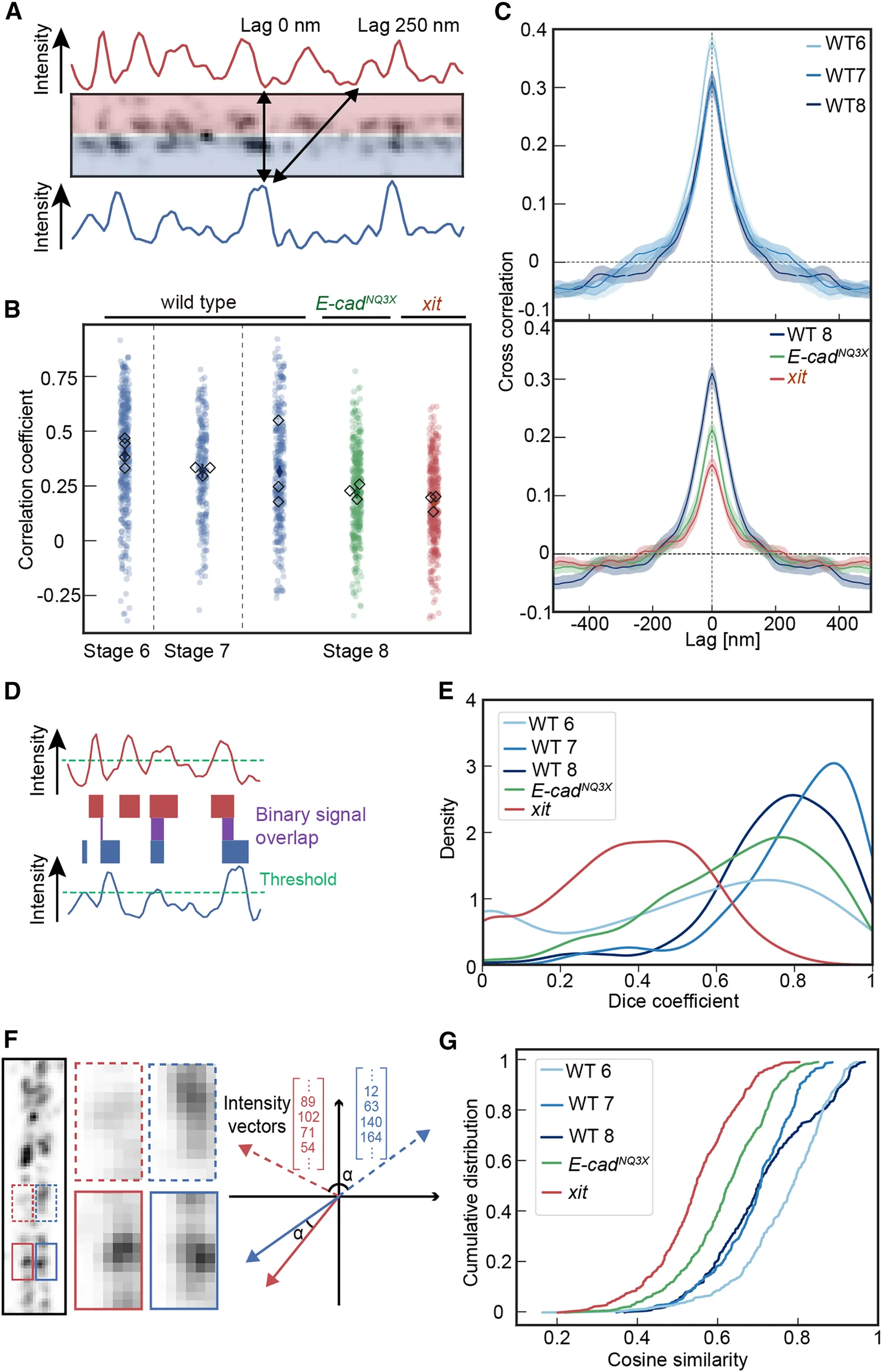
Quantitative comparison of E-cadherin distribution between juxtaposed sides of junctions. (*A*) Depiction of cross correlation analysis. Each straightened junction is split into two regions. Intensities are summed for each junction position, and the resulting intensity traces are analyzed using cross correlation in 1000-nm-long segments. The number of junction segments are WT-stage 6 (four embryos): 370; WT-stage 7 (three embryos): 242; WT-stage 8 (three embryos): 324; *E-cad*^*NQ3x*^ (three embryos): 365; and *xit* (three embryos): 367. (*B*) Correlation coefficients for each stage and mutant. Diamonds are the mean values, and bars indicate the 95% bootstrap confidence interval; each box represents the average value from a single embryo. Differences are statistically significant, except between WT8 and WT7. We note that one embryo in WT stage 8 shows a notably high correlation, which may be an outlier. This could arise from natural variability, differences in imaging region, developmental staging, or recording conditions. The data point is retained to avoid selective reporting but should be interpreted with caution. For additional context, see the independently recorded and analyzed second data set in Figs. 4 and 5. (*C*) Cross correlation function against spatial lag. (*D*) Scheme illustrating the principle of Dice coefficients. (*E*) Kernel density estimate of distribution of Dice coefficients for wild-type stages and mutants calculated from 1000-nm segments (same numbers as in *A*). Original histogram is displayed in Fig. S4*B*. (*F*) Scheme illustrating the principle of cosine similarity between 1000-nm patches from both sides of a junction (same numbers as in *A*). Intuitively, cosine similarity can be thought of as cross correlation between the two-dimensional patches. Intensities are collected in a vector with dimension equal to the number of pixels in each patch. The cosine similarity is defined as the cosine of the angle between those vectors. Patches with similar intensities at each pixel position thus give a value close to 1. (*G*) Distribution of cosine similarities for wild-type stages and mutants.

An obvious question is what fraction of E-cadherin molecules and clusters are engaged in homotypic *trans* complexes. The clusters are very diverse in size and intensity (Fig. 1 *E*), and there is no unique way to define objective criteria for what is regarded a paired cluster. Hence, we turned to computational approaches. We used three distinct measures to assess the degree of pairing and in a more general sense quantified the interdependence of cluster distribution on both junctional sides: 1) cross correlation functions, which quantify the strength and spatial scale with which intensities covary on the two sides of a junction, 2) Dice coefficient, which formalizes the counts of matched clusters disregarding intensities, and 3) cosine similarity, which compares the local spatial pattern of intensities on both sides of a junction (Fig. 3).

Since correlation analysis is based on comparison of the intensity signals, it is a measure of general interdependency and not giving a direct fraction of pairs. Two directly linked E-cadherin clusters could result in a low cross correlation if they differ in the number of molecules. Correlation analysis thus estimates the fraction of paired numbers of molecules but underestimates the fraction of paired clusters.

Cross correlation functions for the profiles (Fig. 3 *A*) were calculated using the natural logarithm of the intensities, because we found that the intensity values are approximately exponentially distributed. In this way an exponential distribution is mapped to a Gaussian distribution and effectively redistributes too much weight from small intensity values to higher intensity values. Correlation functions were calculated in 1-μm-long segments along the junction. Randomly pairing segments results in correlation functions equal to zero for all lags (Fig. S4 *A*). Correlation coefficients were about 0.3 and significantly positive for all three stages in wild-type and with statistically significantly higher values in stage 6 (Fig. 3 *B*). The correlation coefficient was significantly reduced in junctions of both mutants in stage 8. The cross correlation functions showed similar correlation length scales reaching zero approximately at 200 nm in wild-type and mutant junctions (Fig. 3 *C*).

Our analysis reveals a significant correlation in the distribution of E-cadherin between the two sides of the junctions, which changes during development and depends on fully N-glycosylated E-cadherin. We speculate that a higher correlation in stage 6 than stage 8 suggests that initially E-cadherin is preferentially recruited to sites with an intercellular coupling, whereas further on, additional E-cadherin molecules are added for a link to the cortex and without a counterpart on the other side of the junction. Mechanical feedback between cells has previously been shown to facilitate cluster formation and could stabilize junction formation and maturation (64,65).

Secondly, we calculated Dice coefficients, which provide a measure for the fraction of paired clusters independent of intensity (Fig. 3 *D*). The two profiles of the junction traces were binarized by thresholding. The overlap between the two binary signals from each side determines the Dice coefficient by taking twice the overlap divided by the total length of both signals. Using the statistical significance of the profiles as a threshold, we found a high fraction of paired clusters for wild-type stages 7 and 8 (Figs. 3 *E* and S4B). The fraction of paired clusters was lower in wild-type stage 6, the E-cad^NQ3x^ mutant and prominently for *xit* mutants. Using other measures for defining the overlap of the peaks, e.g., Mander M1/M2 coefficient or IoU, we obtained similar relationships (Fig. S4 *C*). In contrast to overlap metrics, the correlation analysis considers the full intensity signal along junctions, penalizing unequal molecular counts and rewarding juxtaposed gaps. Wild-type stage 6 junctions show lower Dice coefficients yet higher correlation compared with stages 7 and 8, thus pointing toward more similar molecular counts and similar cluster location even without perfect overlap.

As a third measure for cluster pairing across the junction, we compared two-dimensional patterns on both sides of the junctions according to cosine similarity (Fig. 3 *F*). To obtain the cosine similarity, intensity values from each patch are summarized in a vector. Multiplication provides the angle between the two normalized vectors, whose cosine gives a value between −1 and +1. Thus, highly matching patterns will have cosine similarities toward 1. We compared regions of 1000 nm × 50 nm to the corresponding but mirrored region of the other side. 50 nm is the range when the average profile has reached its half-point.

For all stages and mutants, we obtained a cosine similarity higher than in randomized images (Fig. S4 *D*), indicating related cluster patterns on juxtaposed sides of the junctions. We found cosine similarities in stage 6 junctions higher than in stages 7 and 8 (Fig. 3 *G*). Stage 8 junctions were largely indistinguishable from stage 7 junctions except for a tail of high similarity patches that significantly deviated from stage 7 junctions, consistent with one embryo showing increased correlation (Fig. 3 *B*). Both mutants, *xit* more than E-cad^NQ3x^, showed less similarity than wild-type between the two sides of the junction as indicated by the left-shifted cumulative distributions curves (Fig. 3 *G*).

Taking together the results from the three methods, our analysis suggests more than 60% of the E-cadherin clusters have a counterpart on the juxtaposed side. We estimated this number from the mean Dice coefficient of wild-type stage 6 embryos, which is 0.6 and, compared with a mean of 0.8 in stages 7 and 8, serves as a lower bound (Fig. S4 *E*). Even if the clusters differ in size, many molecules will be able to form homotypic *trans* complexes. Nevertheless, paired clusters typically differ in size across the junction indicating that they contain a mix of E-cadherin molecules, some engaged in homotypic *trans* complexes and others without a counterpart. During gastrulation the relative proportion of pairing is slightly reduced possibly by incorporation of more unpaired clusters to the junctions. Our analysis indicates an increased number of unpaired clusters in *xit* and E-cad^NQ3x^ mutants, suggesting that N-glycans are critical for pairing.

### The relationship between nanoscopic F-actin and E-cadherin organization at the adherens junction

**Figure 4:**
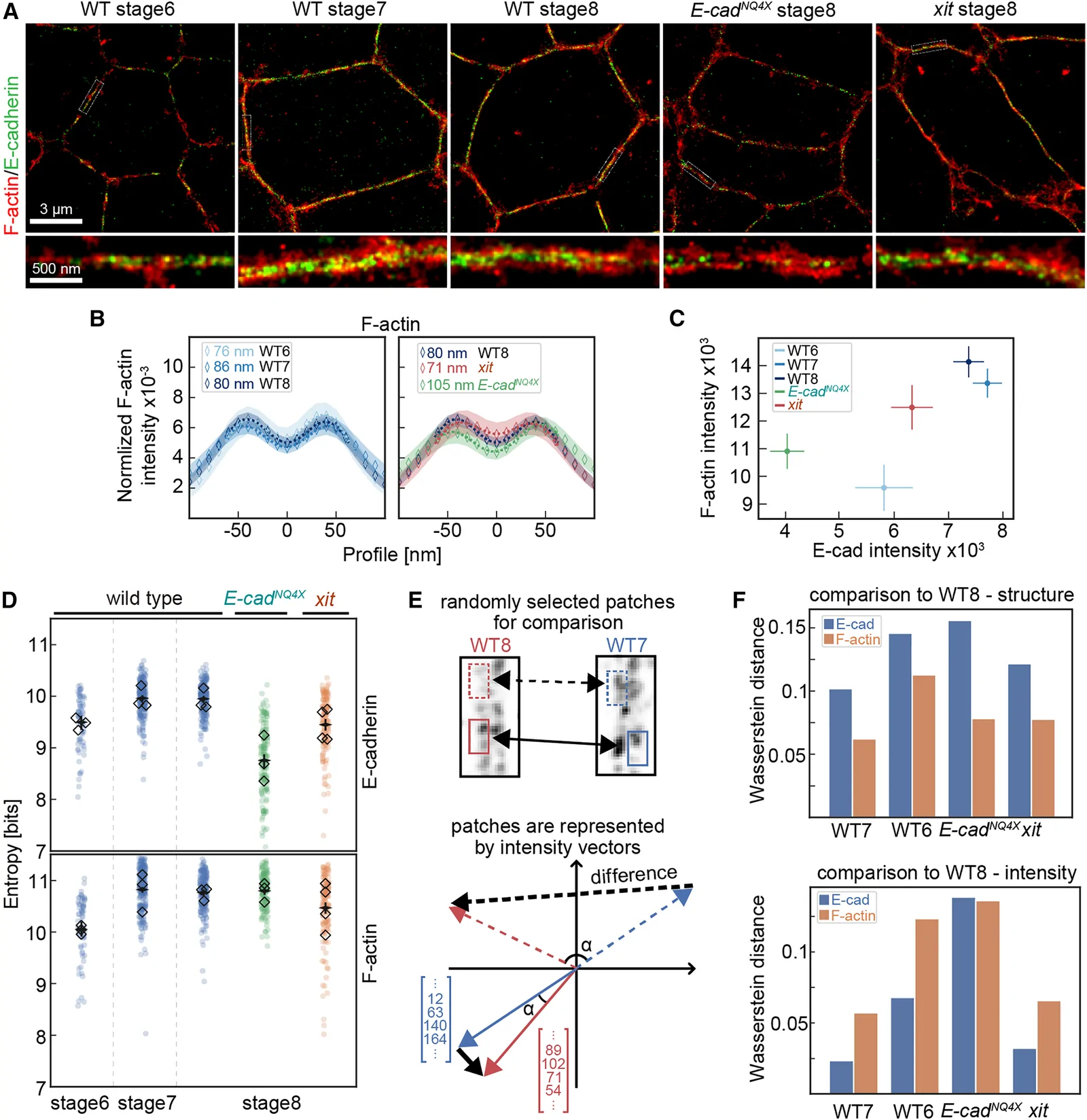
F-actin and E-cadherin double labeling reveals structural differences. (*A*) F-actin (*red*) and E-cadherin (*green*) were simultaneously recorded using DNA-PAINT. Examples show complete cells (scale bar represents 3 μm) and individual junction segments (scale bar represents 500 nm). Both sides of the membrane are clearly visible. (*B*) Averaged normalized F-actin intensity as a function of the profile location displayed as diamonds. The shaded region is the 95% bootstrap confidence interval computed by subsampling average profiles from 1-μm-long segments. The number of segments in this analysis are WT-stage 6 (three embryos): 71; WT-stage 7 (three embryos): 236; WT-stage 8 (three embryos): 216; *E-cad*^*NQ4x*^ (three embryos): 153; and *xit* (four embryos): 137. Dashed lines indicate maximum likelihood fits of two Gaussians to the profile. The estimated width of the membrane corresponding to the distance of the two fitted maxima is given. (*C*) Average F-actin intensity versus average E-cadherin intensity with 95% bootstrap confidence intervals. (*D*) Entropy estimation from 1-μm large junction segments of the F-actin and the E-cadherin spatial pattern. Mean and 95% bootstrap confidence intervals are indicated in black; each box represents the average value from a single embryo. Statistical significance is also given in Fig. S7 and Table S1 in detail. For E-cadherin, WT8-WT7, as well as WT6-*xit* are not significantly different. For F-actin, WT8-WT7-*E-cad*^*NQ4x*^ are not significantly different. (*E*) Depiction of patch comparison method to resolve structural differences in the spatial arrangement of both E-cadherin and F-actin. Patch size was 1000 nm, resulting in the same number of patches as in (*B*). (*F*) Wasserstein distance of patch ensembles in comparison to wild-type stage 8 for E-cadherin (*blue*) and F-actin (*orange*). Smaller bars indicate higher similarity in the spatial arrangement of E-cadherin and F-actin.

We then turn to the analysis of the second data set including wild-type, *xit*, and E-cad^NQ4x^ embryos with F-actin/E-cadherin double-labeled junctions (Fig. 4 *A*). This second data set consists of a few hundred micrometers of junction data from at least three embryos for each stage and genotype (stage 6, three embryos: 41 junctions, 90 μm; stage 7, three embryos: 63 junctions, 270 μm; stage 8, three embryos: 78 junctions, 256 μm; E-cad^NQ4x^, three embryos: 52 junctions, 180 μm; *xit*, four embryos: 56 junctions, 166 μm). To understand the relationship between E-cadherin and associated F-actin, we first analyzed the overall pattern and organization of both molecules at cell junctions. We measured an average distance of the F-actin signal on both membrane sides of 76 nm in wild-type stage 6, 86 nm in wild-type stage 7, 80 nm in wild-type stage 8, 71 nm in *xit* stage 8, and 105 nm in E-cad^NQ4x^ stage 8, which is about 30 nm wider than the E-cadherin signal (Figs. 4 *B* and S5A).

DNA/peptide-PAINT allows us to relate detected blinks to relative differences in molecule numbers across different experiments for F-actin and E-cadherin respectively. We therefore first compared the intensity levels of the measured signals (Fig. 4 *C*). Since the E-cadherin relationship to F-actin is well described in the literature, we expected that relative differences in E-cadherin intensities would largely correspond to relative differences in F-actin. Surprisingly, our data did not support a simple one-to-one relationship across all experiments (Fig. 4 *C*). The E-cad^NQ4x^ F-actin intensity was higher compared with wild-type stage 6 despite 30% less E-cadherin and featuring the strongest overall E-cadherin loss (Fig. 4 *C*). However, our results did confirm a general trend of less E-cadherin corresponding to less F-actin.

We next investigated the F-actin pattern at junctions by calculating cluster size distributions of both E-cadherin and F-actin. The two best fitting cluster size distribution types were again a simple exponential distribution and a power law with exponential cutoff, which were compared using the Bayesian evidence (Fig. S5 *B*–*D*). A power law model with exponential cutoff generally had higher evidence compared with a pure exponential model for both F-actin and E-cadherin cluster size distributions. However, for E-cad^NQ4x^, *xit*, and wild-type stage 6 the F-actin distribution follows a power law with exponential cutoff, whereas the E-cadherin distributions could also be explained by a pure exponential, indicating a possible disconnect in the organization of E-cadherin and F-actin.

To further assess differences in the distribution pattern of both molecules, we applied entropy and patch comparison quantifications as before for the single-channel data set, which were evaluated for 100-nm-sized patches along the junctions (Fig. 4 *D*–*F*). We found that a lower entropy of the E-cadherin signal corresponded to a lower F-actin entropy for all experiments except for E-cad^NQ4x^, indicating a degree of independence of the structural organization of both molecules (Figs. 4 *D* and S7 *B*; Table S1). To obtain a more sensitive readout of structural differences between different experiments, we devised a method to compare the E-cadherin/F-actin layout based on local information from randomly drawn junction patches (Fig. 4 *E*). The difference in the spatial organization between two patches was computed using cosine similarity, as already introduced above (Fig. 3 *F* and *G*). In addition, we took the difference in intensity of two patches into account, given by the lengths of the patch intensity vectors. The total similarity of two patch ensembles can be evaluated by a topological distance metric called Wasserstein distance, where smaller values correspond to more similar structure (Fig. S5 *E*–*I*). This method also provides a readout of junction-to-junction and embryo-to-embryo heterogeneity (Fig. S5 *G* and *H*) (66,67). Generally, heterogeneity decreased with later developmental stages, whereas mutations increased heterogeneity, confirming results of our entropy quantifications (Figs. 2 *H* and 4 *D*) and suggesting a function for those genes in achieving a consistent molecular arrangement. We further compared the structure and intensity of patch ensembles from all experiments with the patch ensemble from wild-type stage 8 as baseline (Figs. 4 *F* and S5 *I*). A larger dissimilarity in the E-cadherin structure and intensity was associated with larger dissimilarity of the F-actin structure and intensity for all experiments except for E-cad^NQ4x^.

All these results indicate that E-cadherin and F-actin are more independently organized in E-cad^NQ4x^ mutants than in wild-type and *xit* embryos. It is not clear why the phenotype in E-cad^NQ4x^ mutants is stronger than in *xit*. However, the *xit* phenotype is complex, as other N-glycosylation targets beside E-cadherin may contribute to the phenotype.

### Correlation analysis reveals an E-cadherin-independent component of nanoscopic F-actin organization

**Figure 5:**
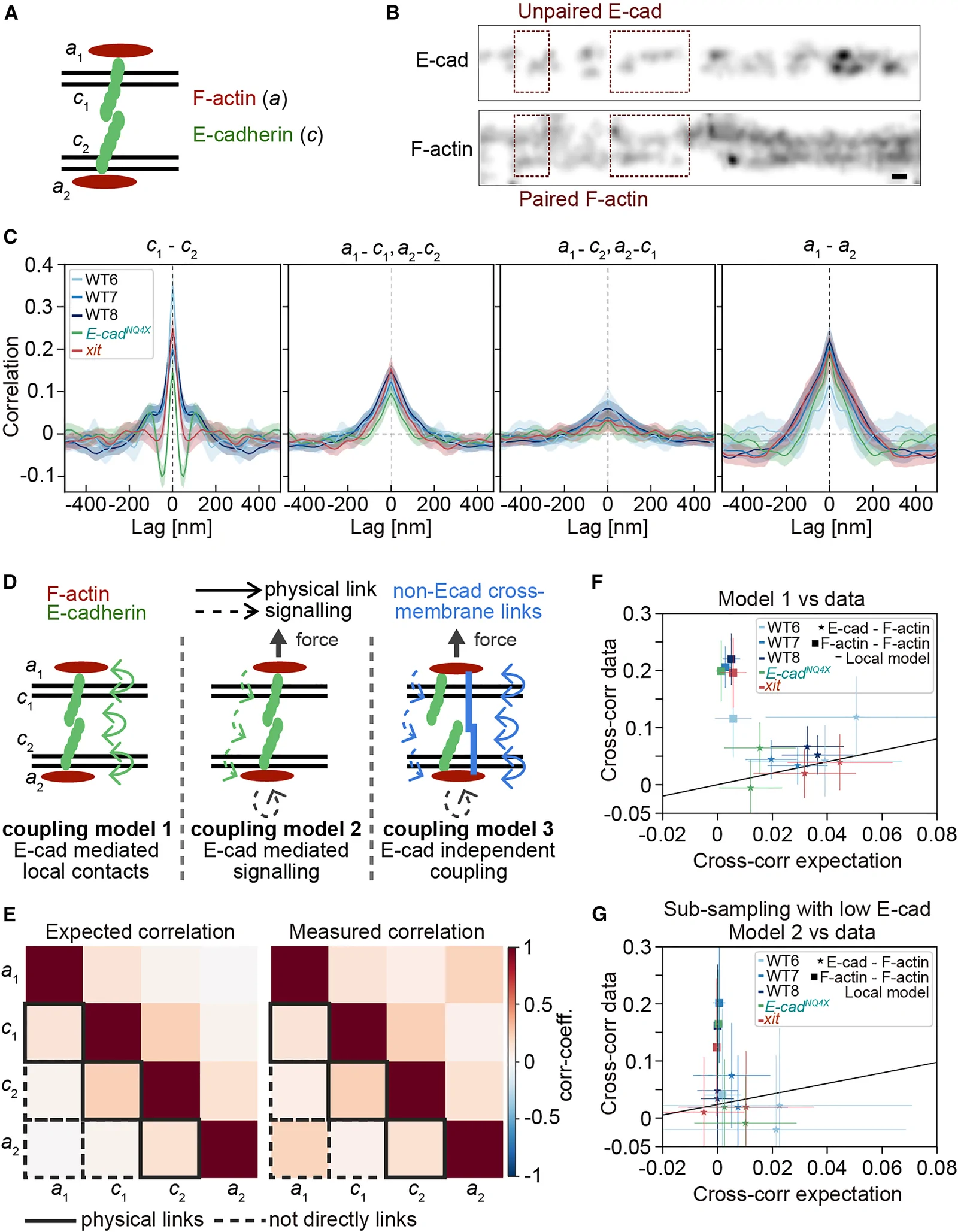
Correlation analysis indicates a degree of independence between E-cadherin and F-actin at the adherens junction. (*A*) Depiction of F-actin signal (a_1_, a_2_) and E-cadherin signal (c_1_, c_2_) at the adherens junction. (*B*) Unpaired and paired E-cadherin (*top*) at an exemplary junction segment and the corresponding F-actin cortex signal (*bottom*), which is mostly paired throughout this example. Scale bar represents 200 nm. (*C*) Cross correlation functions of E-cadherin across the junction (c_1_-c_2_), F-actin and E-cadherin on the same side (a_1_-c_1_, a_2_-c_2_), F-actin and E-cadherin on opposite sides (a_1_-c_2_, a_2_-c_1_), and F-actin on opposite sides (a_1_-a_2_). Colored bands are 95% bootstrap confidence intervals. Correlations were calculated in 1000-nm-long segments. The number of junction segments are WT-stage 6 (three embryos): 71; WT-stage 7 (three embryos): 236; WT-stage 8 (three embryos): 216; *E-cad*^*NQ4x*^ (three embryos): 153; and *xit* (four embryos): 137. (*D*) Depiction of models that could explain the cross-junction organization of F-actin. Model 1: E-cadherin-based local links (*green*) mediate F-actin organization across the junction. Model 2: E-cadherin mediates signals (*green*) that lead to F-actin correlation. In this example a pulling force on one side is transmitted via the E-cadherin *trans* complex leading to recruitment of F-actin on the other side. Model 3: E-cadherin-independent organization of F-actin cortices through either physical links of other membrane proteins or signaling (*blue*). (*E*) Illustration of expected correlation for only local coupling (model 1). The correlation of directly linked signals (*solid black border*) is measured. Assuming model 1, correlations of unconnected signals a_1_-c_2_, a_2_-c_1,_ and a_1_-a_2_ must be mediated by the direct links and can be calculated (*dashed black border*). To the right, we display the actual measured correlations (*dashed black border*). (*F*) Measured correlations of cross-junction F-actin and E-cadherin (a_1_-c_1_, a_2_-c_2_, stars) and cross-junction F-actin (a_1_-a_2_, squares) versus the expected correlation by assuming local coupling only (model 1). (*G*) Same as (*F*) but only using subsampled junction segments that do not share E-cadherin *trans* complexes (*see red regions in B*). This illustrates persistent F-actin correlation despite neither physical links (model 1) nor signaling can be possible (model 2).

To investigate the relationship between E-cadherin and F-actin organization across membrane sides, we calculated correlation functions (Fig. 5 *A*). In the single-channel data set, we only analyzed the cross-membrane correlation between E-cadherin signals (c_1_-c_2_). In the dual-channel data set, we can additionally calculate correlation functions between F-actin on both membrane sides (a_1_-a_2_) as well as the corresponding correlations between F-actin and E-cadherin on the same side (c_1_-a_1_, c_2_-a_2_) and on opposing sides of the junction (c_1_-a_2_. c_2_-a_1_) (Fig. 5 *B* and *C*). By randomly subsampling segments along junctions, we found that all of the above-noted combinations were statistically significant (Fig. S6 *A*). The nonvanishing correlation between F-actin and E-cadherin on the same side (c_1_-a_1_, c_2_-a_2_) quantifies the expected relationship between E-cadherin and F-actin. Intriguingly, however, the correlation of cross-junction F-actin signals was significantly higher, as was the cross correlation of both E-cadherin signals (Fig. 5 *C*). This suggests that the relationship of cortical F-actin between the two sides of the junction is stronger than the intrinsic E-cadherin to F-actin relationship on one side of the junction. We emphasize that this result holds for all data, not just for the E-cad^NQ4x^ mutant.

The degree of F-actin cross-membrane correlation suggests that F-actin cortices of neighboring cells are not independent from each other. To understand whether E-cadherin could be solely responsible for intercellular F-actin organization or whether other factors might be at play, we undertook a thought experiment that could be directly tested using our data.

First, we assume a model that allows only local coupling between all constituents (Fig. 5 *D*). In this model, F-actin (a_1_) would bind physically (for instance through alpha-catenin) to E-cadherin (c_1_), which is linked to E-cadherin on the other membrane, (c_2_) which is again physically linked to F-actin (a_2_). The correlations between cross-junction F-actin (a_1_-a_2_) and cross-junction E-cadherin and F-actin (a_1_-c_2_, c_1_-a_2_) can therefore only be mediated through intermediate proteins as they do not share direct links. Under these assumptions, we can calculate the expected correlation between F-actin on both junction sides (a_1_-a_2_) from the known measured direct correlations between both E-cadherin signals (c_1_-c_2)_ and E-cadherin-F-actin on each side (a_1_-c_1_, c_2_-a_2_). Comparing the expected correlation with the calculated correlations from our data showed that the cross-membrane F-actin correlation (a_1_-a_2_) was far higher than expected (Figs. 5 *E* and *F*, and S6B). This shows that the observed F-actin cross correlation cannot be caused by just local linking with E-cadherin. In contrast, cross-junction F-actin and E-cadherin correlations (a_1_-c_2_, c_1_-a_2_) behaved exactly as expected, indicating that those proteins are not organized by more than just direct links.

A second model that could explain how E-cadherin mediates F-actin cross correlation is via nonlinear signal transmission. For example, mechanical forces could be transmitted through transmembrane E-cadherin complexes and lead to recruitment of F-actin on the other side (Fig. 5 *D*). To test for this possibility, we calculated all cross correlations only at parts of junctions with E-cadherin gaps on both membrane sides. Since no E-cadherin *trans* complex exists at those junction segments, signals could obviously not be transmitted. We therefore expected a significant drop in the F-actin cross correlation. However, we found that F-actin cross correlation persisted at parts without E-cadherin bridges, except for wild-type stage 6 embryos (Figs. 5 *G* and S6 *C*). This indicates a role of E-cadherin for organization of intercellular F-actin cortices during early stages of development. In later stages, however, our results suggest a lower degree of E-cadherin dependence for the cross-junctional organization of F-actin.

## Discussion

We managed to resolve E-cadherin clusters and their relation to the F-actin cortex both laterally as well as across adherens junctions by applying DNA/peptide-PAINT nanoscopy on *Drosophila* embryos. Our imaging of adherens junctions in the epidermis in whole-mounts with a resolution below 40 nm revealed multiple unexpected results, namely a significant fraction of unpaired E-cadherin clusters and an independence of E-cadherin and cortical F-actin. In order to mitigate labeling inefficiencies that limit every labeling-based study and that may introduce some bias in the observed absolute molecule numbers, we designed (partially novel) statistical approaches and quantifications. This allowed us to draw conclusions based on relative numbers under the assumption that these labeling inefficiencies are homogenous. Due to DNA-PAINT’s capability for single-molecule localization, we only expect very small clusters, containing a few molecules, to be underrepresented in our measurements. In denser clusters, fluorescence intensity could be nonlinearly suppressed. Although we would still detect those large clusters, estimations based on absolute molecule numbers might be biased toward lower values as a result, such as the location of the exponential cutoff in the cluster size distribution. However, on average, this would not affect similarity quantifications, like correlation, since the effect would occur independently on both junction sides. Additionally, the lower axial resolution of our setup may lead to an overestimation of paired clusters and correlations, as clusters in different depths are projected onto the same image. Therefore, our estimates should be viewed as an upper bound of the actual number of clusters engaged in *trans* binding.

*Drosophila* germband extension is largely driven by local neighbor exchanges, with actively remodeling cell junctions. Our findings should be considered in this dynamic context: cell contacts must balance short-term elasticity and stability with plasticity on longer timescales to facilitate tissue fluidity and rearrangements. E-cadherin has been shown to play a crucial role in regulating junction constriction and intracellular force generation, interacting with F-actin via proteins such as α-catenin (7). Together with nonmuscle myosin II, these components generate the forces driving junction constriction and are continuously interacting and rearranging. Static epithelia, in constant mechanical equilibrium, may benefit from a distinct molecular organization at cell interfaces that focus more on stability. Our results provide insights into the nanoscopic molecular organization that underlies mechanical signaling, cell interactions, and adhesion during epithelial morphogenesis.

Being able to distinguish the juxtaposed sides, we analyzed the distribution pattern of E-cadherin separately for both sides. Importantly, despite the stochastic spatial organization, following a power law with exponential cutoff in matured junctions, a good fraction of clusters had counterparts on the juxtaposed side, indicating potential homotypic *trans* complexes. Both the spatial pattern as well as the degree of pairing changes during development and depends on N-glycosylation of E-cadherin.

However, we also observed that many junction segments were without an apparent E-cadherin *trans* complex connecting the two membranes. This is unexpected, since E-cadherin molecules are assumed to form *trans* complexes constitutively and as long as binding partners are available, given the binding energy of the complex. Apparently preconditions exist for *trans* binding in addition to extracellular Ca^2+^ in the millimolar range. Multiple parameters are conceivable. 1) Posttranslational modifications in the extracellular part that would act similarly to Ca^2+^ and promote a conformation allowing trans binding. 2) Regarding mechanical load on the intracellular domain, E-cadherin complexes that form *cis* clusters without being exposed to mechanical load may become blocked for *trans* interaction. In contrast E-cadherin molecules under mechanical load may first engage in a *trans* interaction and afterward form *cis* clusters with other *trans* complexes leading to paired clusters.

The highest correlation of the E-cadherin signals was found in early stages of development, which is consistent with the notion that initially punctate adherens junctions form, which mature toward the full zonula adherens junction with uniformly distributed E-cadherin (21). In addition, F-actin correlations in wild-type stage 6 could be explained purely by E-cadherin-based signaling, suggesting a crucial role of E-cadherin for force-dependent junction maturation in early development. In this light, E-cadherin’s primary function could be for junction formation and rearrangement, whereas stability and connection to the intracellular F-actin cortices are given to other molecules such as Nectin (68).

We discovered that F-actin was strongly correlated across the junction, in fact even more than E-cadherin with F-actin in the same junction. This suggests that an E-cadherin-independent mechanism may be involved in the intercellular coordination of F-actin cortices. If true, this result would redefine our current understanding of the molecular organization of adherens junctions and have implications for force regulation and force-dependent adhesion in epithelial morphogenesis. In addition to testing this hypothesis computationally, we also provided direct evidence using a novel E-cad^NQ4x^ mutation, which had the strongest effect on E-cadherin of all assays tested, but it affected F-actin to a much lesser extent. We stress that our results do not suggest complete independence between E-cadherin and F-actin, as can for example be seen from a drop of F-actin correlation in case of low E-cadherin signal (Fig. 5 *G*); they rather point to an additional mechanism organizing F-actin.

A conceivable explanation for the intercellular organization of F-actin is that one or more transmembrane adhesion molecules interact redundantly with E-cadherin. Recently, a study in human intestinal epithelia demonstrated that the main fraction of circumferential F-actin cortex assembles apical of E-cadherin clusters where the connection is mediated by Nectin, an adhesion protein with Ig domains, via the F-actin binding protein afadin (16). Echinoid (Ed), the Nectin homolog in *Drosophila*, colocalizes with E-cadherin at adherens junctions (68). Echinoid function has remained unclear as Ed mutant embryos contain apparently normal junctions (68). A second candidate is the peripheral membrane protein ZO-1 homolog, Polychaetoid (Pyd) (69,70). Similar to Echinoid, Polychaetoid colocalizes with E-cadherin at embryonic junctions (71). Pyd serves a function in cell rearrangement in the epidermis, but *pyd* mutants display apparently normal E-cadherin distribution on microscopic scale at least (71).

For stable adhesion, one may expect a uniform distribution of the “glue” molecules along the interface to distribute forces equally among many connections. The apparent stochastic spatial pattern of E-cadherin clusters and cortical F-actin shows that the underlying processes that govern E-cadherin and F-actin association at the membrane are more complex. To better understand the underlying laws that govern the molecular dynamics responsible for the observed patterns a thorough quantitative grasp of their spatial arrangement is needed for comparison to predictions of mathematical models and across different experiments.

In this study, we introduced the entropy and patch comparison methods that provide direct readouts of similarity of spatial patterns. Both methods are segmentation free and can be generally applied to all kinds of spatial fluorescence signals in biology. Entropy is easy to compute and quantifies the generalized variance of arbitrarily complex distributions, signifying an increasingly homogeneous distribution with larger values. Since entropy is an established metric of organization in complex systems, we expect it to provide sensitive information on spatial arrangements in a vast variety of contexts. The patch comparison method, on the other hand, quantifies local differences in spatial patterns and does not rely on coarse-graining summary statistics. Global similarity of two spatial distributions can be obtained using the Wasserstein distance from optimal transport theory, providing a scalar and simple readout. In other words, two patterns are considered to be similar if the overall differences of their local spatial structure are small. The strength of the patch comparison method lies therein that the actual local structure is taken into account while still producing a simple comparison of the global pattern. One of our use cases was quantifying junction-to-junction and embryo-to-embryo heterogeneity. Interestingly, the extent and functional significance of biological heterogeneity remain poorly defined, and there is no consensus on how best to quantify it. By directly measuring junction-level variability in this study, we provide a quantitative benchmark that helps clarify these questions. Our approach offers a general framework for assessing variability in diverse developmental contexts, contributing to a deeper understanding of robustness and heterogeneity in morphogenesis.

Finally, our study demonstrates the potential of DNA-PAINT for investigating the nanoscopic molecular organization of epithelial tissue in vivo during development. Our setup allows to resolve E-cadherin at juxtaposed sides of membranes at adherens junctions with simultaneous co-imaging of F-actin. Future studies will utilize two or potentially three channels for differentially tagged components such as E-cad-GFP and α-catenin-halo or myosin-II-SNAP to further dissect the arrangement and stoichiometry of adherens junctions. Especially interesting will be the structure of adhesion proteins and their relation to mechanics and forces at the junction. For example, in a simple model, one may expect more paired clusters and a stronger correlation between E-cadherin and cortical F-actin in mechanically challenged junctions.

Additionally, cell and junction mechanics is known to be regulated by genetic signaling, which establishes planar cell polarity during germband extension. Junctional MyoII exhibits a planar-polarized distribution, being enriched at DV-oriented junctions (along the dorsal-ventral axis) rather than AP-oriented junctions (along the anterior-posterior axis) in the lateral epidermis. This anisotropic localization results in junctions experiencing different levels of tension depending on their orientation. Future studies could therefore investigate nanoscopic organization of the components of the adherens junctions for different junction orientations and use our quantitative tools to detect differences, thereby providing crucial insights into the interaction between mechanical forces and the nanoscale organization of adherens junctions.

Another obvious extension of our study could be to investigate molecular organization at tricellular junctions, since we here only focused on bicellular junctions. We expect that DNA-PAINT will complement existing super-resolution methods because of its unique ability for absolute molecule counting, ease of registration free multichannel recording, and importantly tissue imaging.

In summary, this study provides novel insights into the nanoscale organization of adherens junctions during *Drosophila* morphogenesis. Future studies will introduce further junction-associated proteins into the nanoscopic picture, address the stoichiometry of the E-cadherin complexes in situ, and relate the structure to the mechanical load on junctions.

## Data and code availability

All the data supporting the findings of this study are available within the paper. Raw image data are available upon request. The custom codes used to process images and analyze data are available upon request.

## Acknowledgments

We acknowledge A. Neef, M. Wibral, S. Rizzoli, M. Sternbach, Z. Stawyskyj, J. Vogel, K. Willig, and D. Zwicker for discussions. We acknowledge service support from the Bloomington Drosophila Stock Center (supported by NIH P40OD018537). This work was in part supported by the 10.13039/501100001659Deutsche Forschungsgemeinschaft (DFG GR1945/15–1, GR1945/17-1, WO1489XX), equipment grant (INST1525/16-1 FUGG), the 10.13039/501100001663VolkswagenStiftung (A129197, ZN2632), and the Cluster of Excellence Multiscale Bioimaging (MBExC).

## Author contributions

J.G. and D.K. conceived the study. M.H. and F.W. conceived computational methods. D.K. and J.G. conducted the experiments. Y.Z., N.Z., M.H., and Z.L. segmented junctions. M.H. developed algorithms and performed analysis. G.S., J.H.R., E.S.A., and D.St.J. developed and established experimental methods, hardware, and computational procedures for nanoscopy. M.H., D.K., F.W., and J.G. analyzed the results. M.H., F.W., J.G., and D.K. wrote the manuscript with comments from all authors.

## Declaration of interests

The authors declare no competing interests.

## Supporting material

Document S1. Figures S1–S8 and Table S1

Document S2. Article plus supporting material
