# Supplementary material for: DNA-PAINT resolves E-cadherin-independent cross-junctional F-actin organization in Drosophila embryonic tissue
Source: Biophys J. 2026 Jan 17;125(5):1211–29. doi: 10.1016/j.bpj.2026.01.026 (PMC13351991; doi:10.1016/j.bpj.2026.01.026)
Supplement: Document S1. Figures S1–S8 and Table S1 [file mmc1.pdf]

Biophysical Journal, Volume *125*

Supplemental information

DNA-PAINT resolves E-cadherin-independent cross-junctional F-actin organization in *Drosophila* embryonic tissue

Matthias Häring, Yuanshu Zhang, Na Zhang, Edward S. Allgeyer, Jennifer H. Richens, George Sirinakis, Zhiyi Lv, Daniel St Johnston, Fred Wolf, Jörg Großhans, and Deqing Kong

Supplemental information

Supplemental Figures

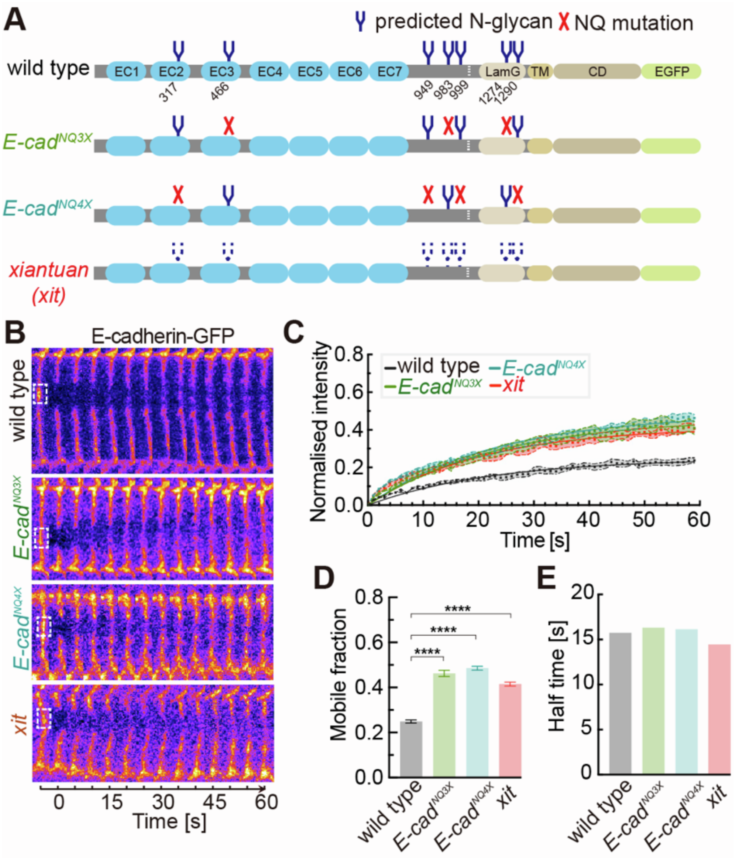

Figure S1 Effects of N-glycosylation mutations on mobile fraction of E-cadherin.
(A) Schematic depiction of N-glycosylation mutation sites in the E-cadherin^{NQ} alleles.
(B-E) E-cadherin-GFP fluorescence recovery after photobleaching in stage 8 embryos from wild type, *E-cad*^{NQ} and *xit* mutants.
(B) Kymograph of E-cadherin-GFP pre- and post-bleaching. The white dashed box indicates the region of photobleaching.
(C) Normalized fluorescence intensity. Measurements from 15 experiments with mean with SEM. Lines indicate one-phase association fitting.
(D, E) E-cadherin-GFP mobile fractions (D), and mean recovery times after photobleaching (E). N = 19 junctions from more than three embryos in each genotype.
Unpaired two-sided *t*-test estimate the *p* value, **** $p<0.0001$.

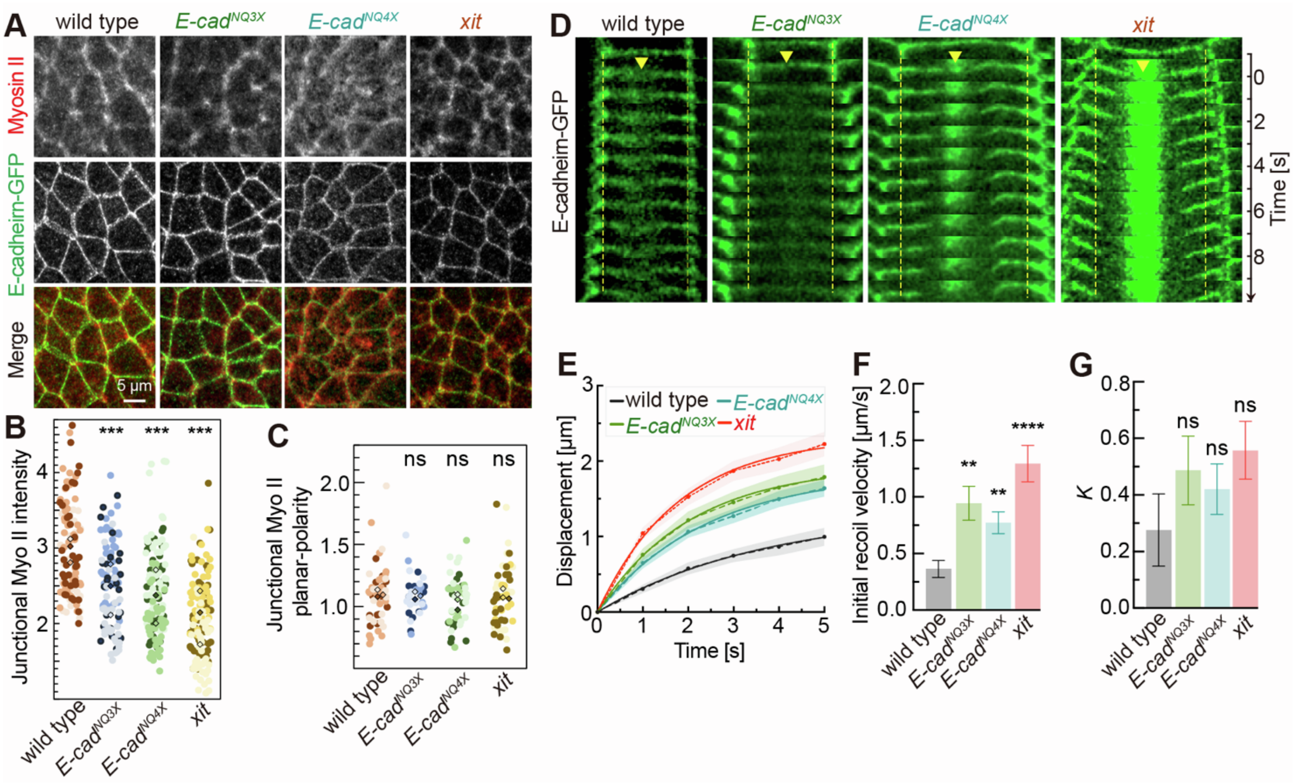

Figure S2 Effects of N-glycosylation mutations on cell mechanics.

(A) Representative images of Non-muscle myosin II (detected by Zipper antibody) together with E-cadherin-GFP (detected by GFP antibody). Scale bar 5 μm.

(B, C) Quantification of junctional Myosin II intensity (B) and planar polarity (C) from 3 embryos in each genotype. Each dot represents a cell junction, with the same color indicating the same embryo, and the square represents the average value from a single embryo.

(D) Kymographs of E-cadherinGFP labelled junctions from laser microdissection experiments. Time is directed from top to bottom and aligned to the time of the junction cut event, also indicated by the yellow triangle. Dashed lines indicate the original distance of adjacent 3x vertices. The green spots in the image at the cutting location are background noise caused by the UV-laser during the cutting process.

(E) Vertex displacement versus time plot for junction cuts. Dots represent the mean with SEM of displacement of junction cuts. Solid lines represent the one-phase association fitting of experimental data. N=20 junctions from 20 embryos in each genotype.

(F) Mean with SEM of initial recoil velocities from the fit of the curves in (E).

(G) Friction coefficient from the fit of the curves in (E).

Unpaired two-sided *t*-test estimate the *p* value, *** p<0.01,**** p<0.0001, ns, no significance.

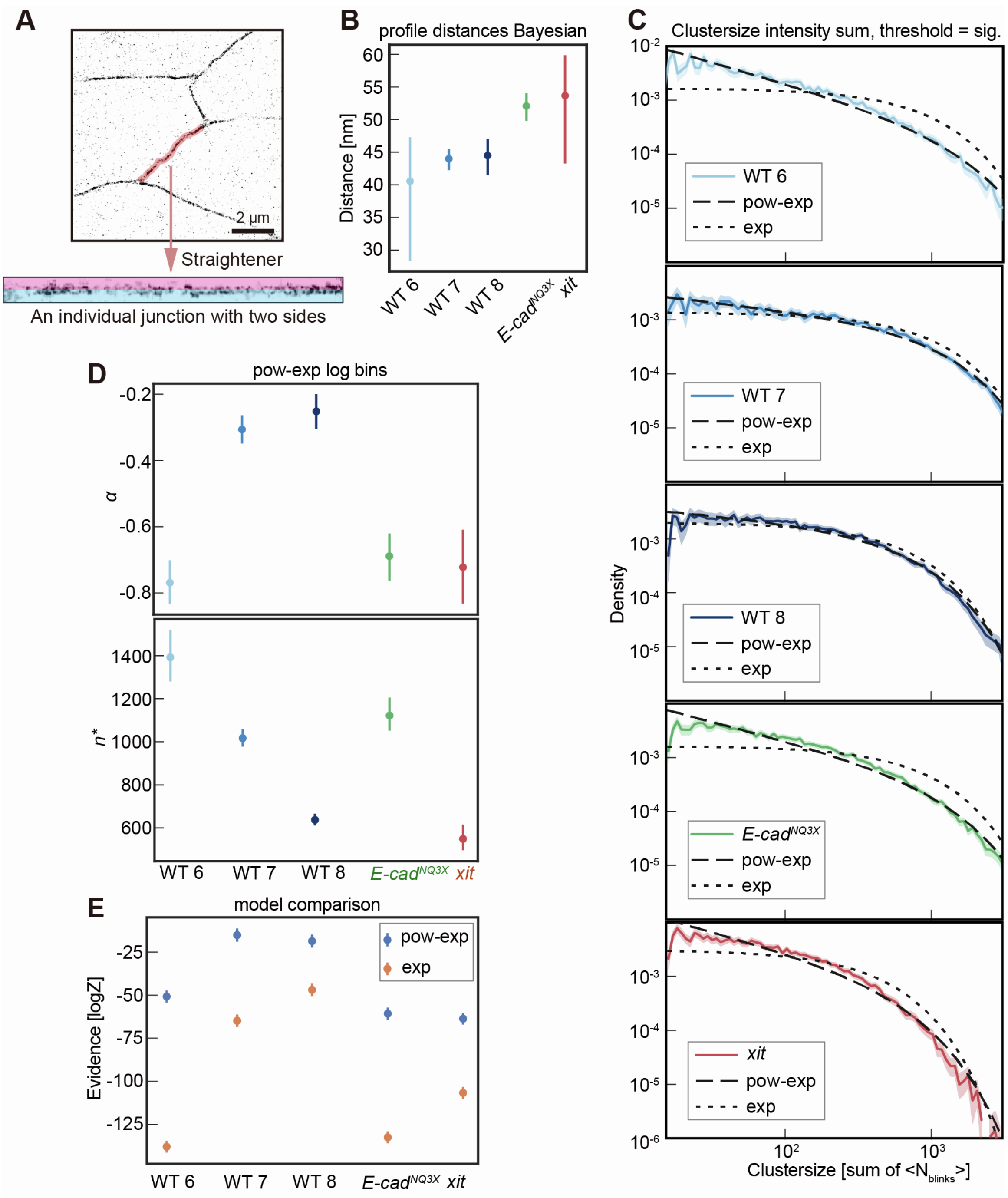

Figure S3 E-cadherin cluster-size distribution.

(A) Manually segmented junctions were straightened and split into two sides.

(B) Bayesian estimate of the two Gaussians.

(C) Cluster-size distribution with inferred maximum posterior exponential or power law models. The cluster size equals to the integrated intensity in a segmented pixel group. Shaded error regions are 95% bootstrap confidence intervals.

The developmental stages and genotypes are indicated. The number of clusters are WT-stage 6 (4 embryos): 9492, WT-stage 7 (3 embryos): 10552, WT-stage 8 (3 embryos): 11255, *E-cad*^{NQ3x} (3 embryos): 17518, *xit* (3 embryos): 11927.

(D) The inferred parameters for the power law scaling α and n^*. Error bars indicate the credible interval, which is the 95% confidence interval of the posterior distribution.

(E) Bayesian model comparison of the evidence between the power law model with exponential cutoff and the pure exponential distribution. Error bars indicate the uncertainty about the evidence at the end of the nested sampling run.

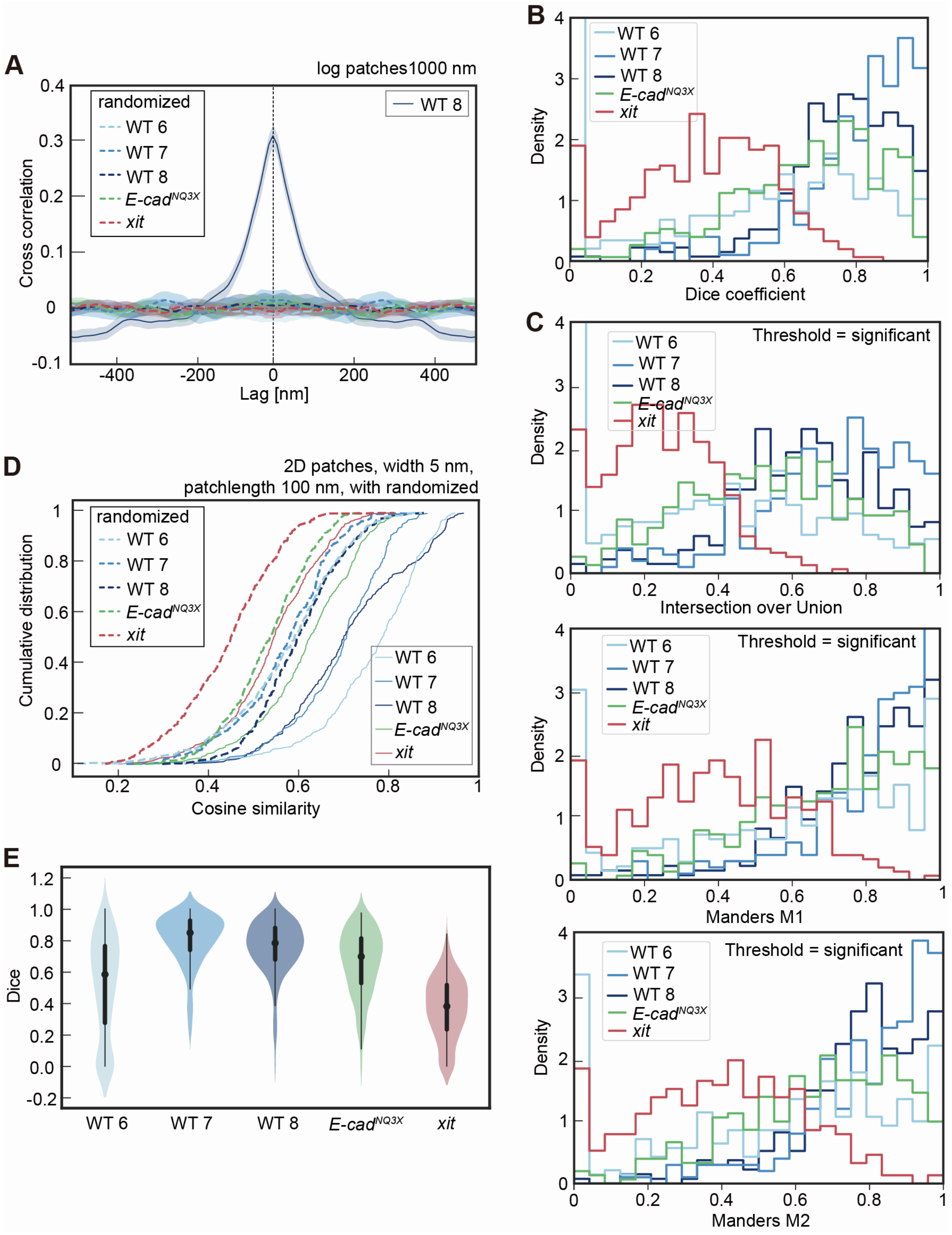

Figure S4 Quantification for the paired and unpaired E-cadherin clusters at adherens junctions.

(A) Cross correlation functions of randomized data in comparison to wild type stage 8.

(B) Distribution of Dice coefficients for wild type stages and mutants.

(C) Variations of overlap quantification: Manders and Intersection over Union. Distributions for wild type stages and mutants.

(D) Cosine analysis with randomized data (dashed lines) in comparison to original data (solid lines).

(E) Data in (B) shown as violin plot to make the location of the mean visually apparent. Data in (A)-(E) has been analyzed from 1000 nm long segments. The number of junction segments are: WT-stage 6 (4 embryos): 370, WT-stage 7 (3 embryos): 242, WT-stage 8 (3 embryos): 324, *E-cad*^{NQ3x} (3 embryos): 365, *xit* (3 embryos): 367.

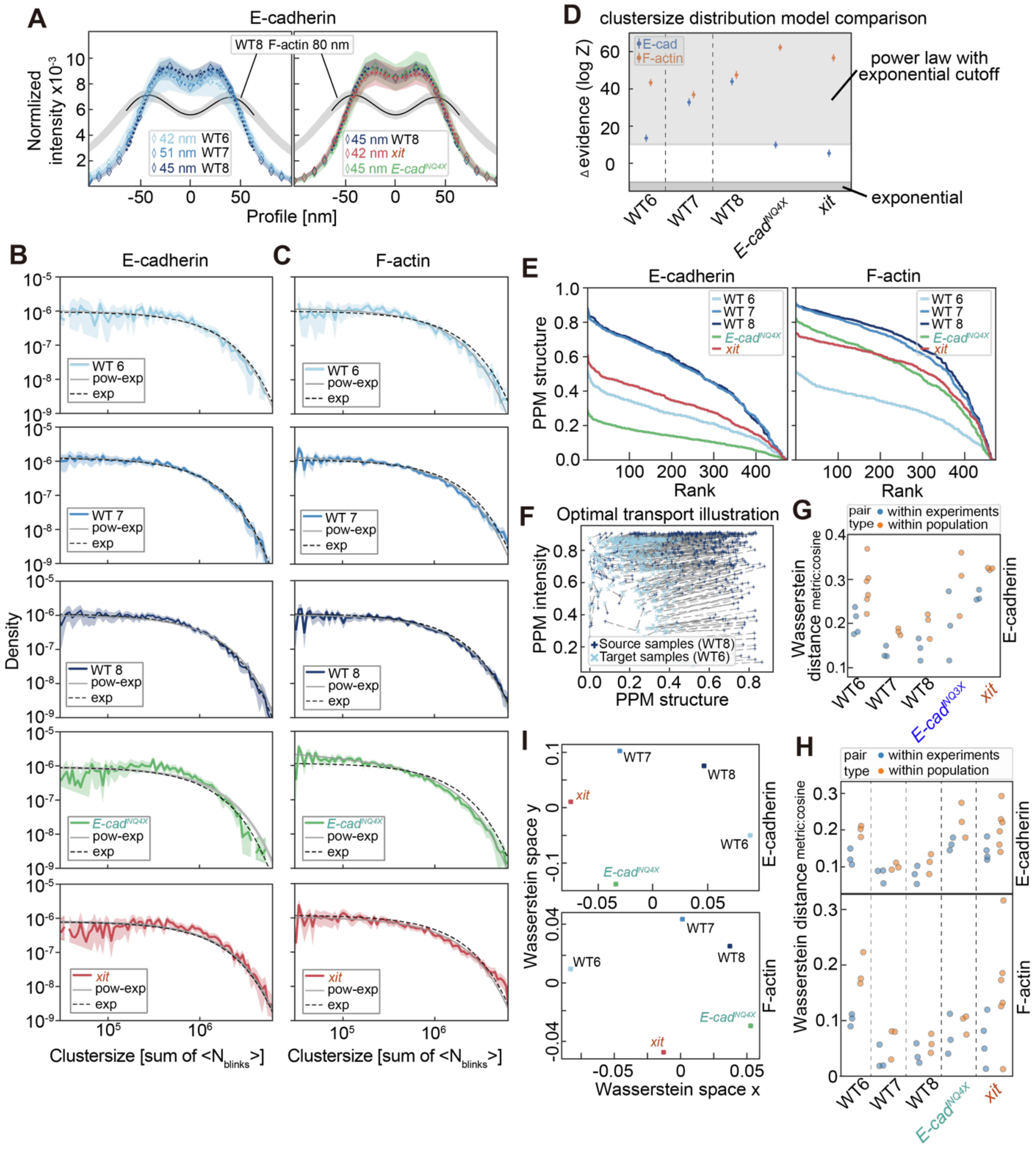

Figure S5 F-actin and E-cadherin organization.

(A) Intensity profiles of the double label data with indicated width between the junction sides. Normalized mean of the signal (diamond) with bootstrap 95% confidence interval (shaded region). Dashed lines indicate maximum likelihood fits of two gaussians to the profile.

(B) Cluster size distribution of E-cadherin with indicated bestfit powerlaw model with exponential cutoff and pure exponential distribution. The number of detected clusters are: stage 6 (3 embryos): 1176, stage 7 (3 embryos): 5400, stage 8 (3 embryos): 4433, E-cad^{NQ4x} (3 embryos): 1852, *xit* (4 embryos): 1863.

(C) Same as (B) but for F-actin. The number of detected clusters are: WT-stage 6 (3 embryos): 2129, WT-stage 7 (3 embryos): 8468, WT-stage 8 (3 embryos): 7366, *E-cad*^{NQ4x} (3 embryos): 5769, *xit* (4 embryos): 3773.

(D) Model comparison of a power law distribution with exponential cutoff and a purely exponential distribution for the cluster size distribution of both E-cadherin (blue) and F-actin (orange). Given is the difference in log evidence of the two models. The gray regions indicate for which log-evidence difference a model has higher statistical support from the experimental measurements.

(E) Rank distributions of patch probability masses by comparing patches with the full ensemble of the respective genotype. A high patch probability mass indicates that there are many patches in the ensemble that are similar. Lower probability mass indicates uniqueness of a patch.
(F) Illustration of optimal transport and Wasserstein distance. Points of two ensembles, here WT stage 8 (dark blue) and WT stage 6 (light blue), are shifted such that they coincide. Gray dashed lines indicate with points of the two distributions are connected. The length of those lines indicates the distance of two patches. The Wasserstein distance is the sum of all distances.
(G, H) Analysis of junction-to-junction (blue dots) and embryo-to-embryo (orange dots) heterogeneity. Results for dataset 1 are shown in (G) and results for dataset 2 are shown in (H). Wasserstein distances were calculated from junction patches within a single experiment (image recording), generated using random subsampling (blue dots), and Wasserstein distance among the population belonging to the same experiment class, for example among wild type stage 8 (orange dots). Heterogeneity goes down with later developmental stages and mutations generally increase heterogeneity, consistent with the entropy quantification (Figures 2H and 4D).
(I) Similarity between all genotypes is displayed in Wasserstein space. Wasserstein distances between all respective patch ensembles were calculated and afterward arranged in the 2d space such that the individual distances are optimally represented.
Except for (B)-(D) all data in this figure has been analyzed in 1000 nm long segments. The number of junction segments are: WT-stage 6 (3 embryos): 71, WT-stage 7 (3 embryos): 236, WT-stage 8 (3 embryos): 216, *E-cad*^{NQ4x} (3 embryos): 153, *xit* (4 embryos): 137.

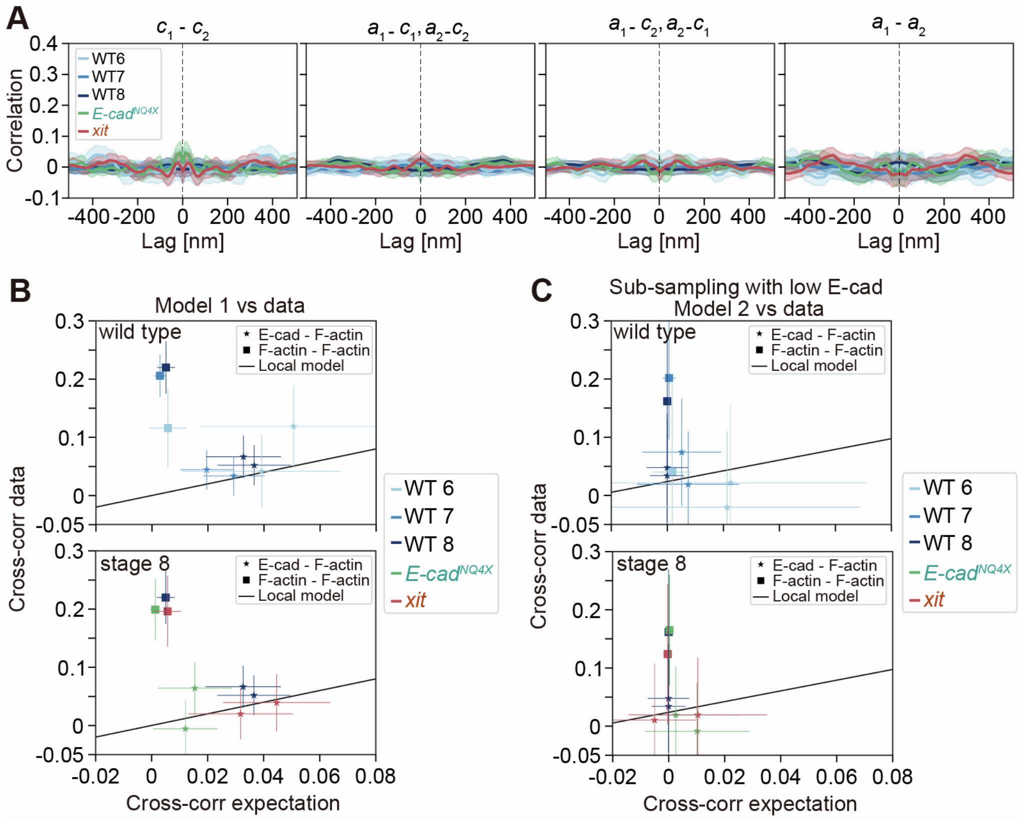

Figure S6 Independence of E-cadherin and F-actin.

(A) Cross correlation functions of randomized data in comparison for all combinations of the 4 intensity signals. Correlations were calculated in 1000 nm long segments. The number of junction segments are: WT-stage 6 (3 embryos): 71, WT-stage 7 (3 embryos): 236, WT-stage 8 (3 embryos): 216, E-cad^{NQ4x} (3 embryos): 153, *xit* (4 embryos): 137.

(B, C) Same as Figure 5F and G but split for better visibility.

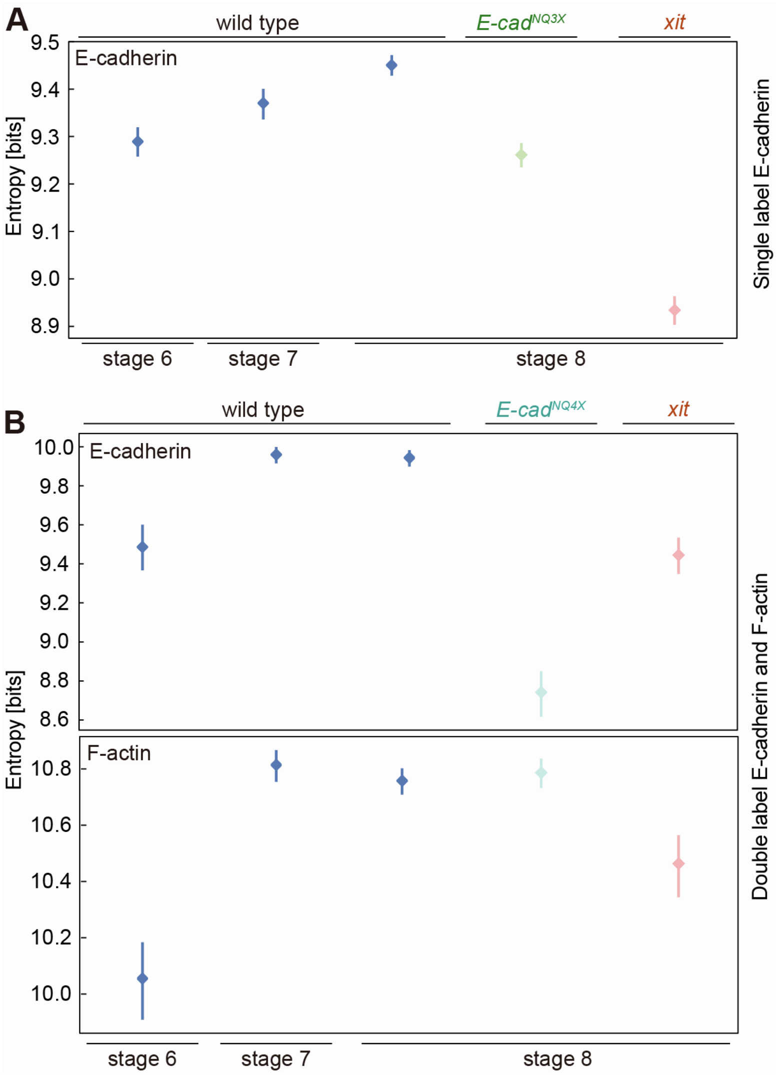

Figure S7 Illustration of 95% confidence interval for entropy analysis.

(A) The 95% confidence interval for entropy analysis of E-cadherin from E-cadherin single label dataset (Figure 2H).

(B) The 95% confidence interval for entropy analysis of E-cadherin and F-actin from E-cadherin-F-actin double label dataset (Figure 4D).

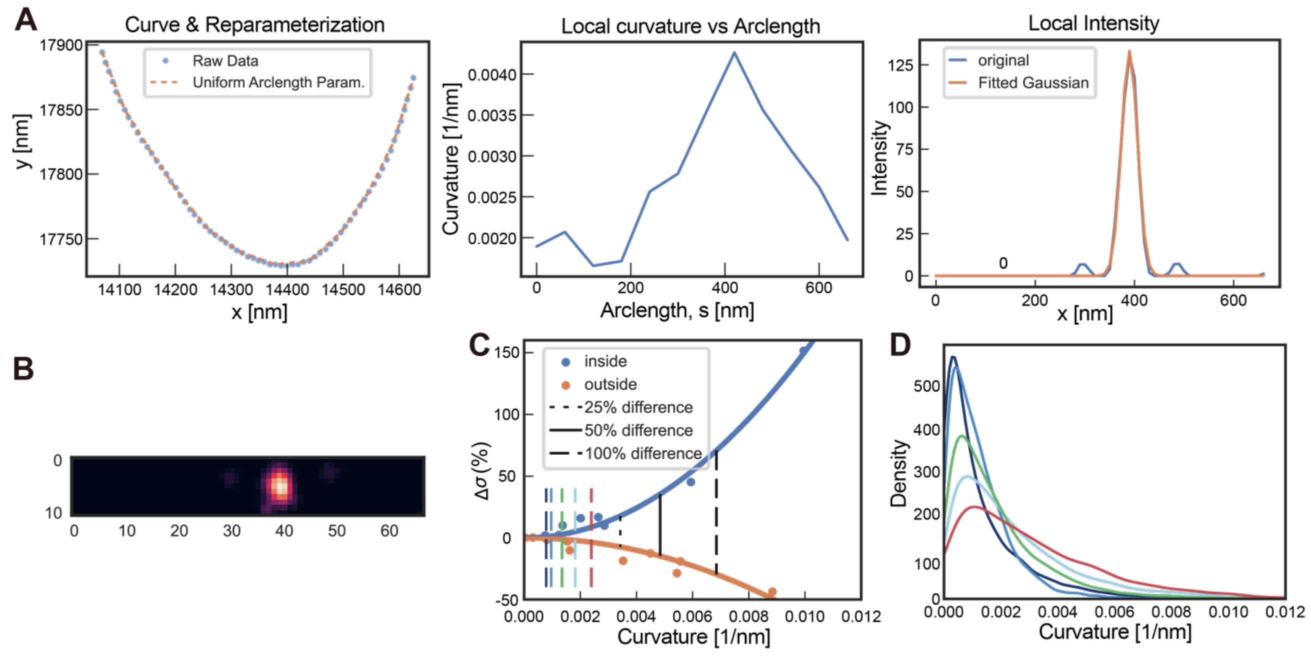

Figure S8 Evaluation of FIJI‘s “straighten“ operation.

(A) Example of evaluation procedure which consists of parameterizing the segmented line by the arclength of the curve (left), calculating the local curvature along the arclength (middle) and then fitting a Gaussian to local intensity values of the resulting clustering after ”straighten" operation (right). The standard deviation of the Gaussian serves as measure of deformation strength.

(B) Example of straightened single cluster used for analysis.

(C) Measured difference of the standard deviation after straightening in comparison to original, not straightened, value (dots). Lines represent best-fit quadratic functions and colors denote sign of curvature (cluster ”inside” or ”outside” of curved segmented line). The dashed vertical colored lines represent mean values of curvature distributions (see (D)) from different stages and mutations (dark blue WT8, middle blue WT7, light blue WT6, green NQ mutations, red *xit* mutation).

(D) Distribution of measured local curvature from all segmented junctions. The number of evaluated junctions are: WT-stage 6 (3 embryos): 71, WT-stage 7 (3 embryos): 236, WT-stage 8 (3 embryos): 216, E-cad^{NQ4x} (3 embryos): 153, *xit* (4 embryos): 137.

Supplemental tables

Supplemental table1 P-values of entropy analysis.

Single label E-cadherin

Entropy of E-cadherin

	WT 8	WT 7	WT 6	*E-cad*^{NQ3X}	*xit*
WT 8	1.00	4.08e-05	9.22e-16	5.47e-25	1.59e-108
WT 8	4.08e-05	1.00	6.01e-04	4.31e-07	2.03e-62
WT 6	9.22e-16	6.01e-04	1.00	0.17	2.91e-51
E-cad^{NQ3X}	5.47e-25	4.31e-07	0.17	1.00	2.31e-50
xit	1.59e-108	2.03e-62	2.91e-51	2.31e-50	1.00

Double label E-cadherin and F-actin

Entropy of E-cadherin

	WT 8	WT 7	WT 6	*E-cad*^{NQ4X}	*xit*
WT 8	1.00	0.59	1.69e-17	1.88e-66	1.05e-23
WT 7	0.59	1.00	2.46e-18	6.18e-71	1.53e-25
WT 6	1.69e-17	2.46e-18	1.00	4.83e-13	0.60
E-cad^{NQ4X}	1.88e-66	6.18e-71	4.83e-13	1.00	1.97e-17
xit	1.05e-23	1.53e-25	0.60	1.97e-17	1.00

Entropy of F-actin

	WT 8	WT 7	WT 6	*E-cad*^{NQ4X}	*xit*
WT 8	1.00	0.13	2.94e-27	0.43	7.51e-08
WT 7	0.13	1.00	2.93e-26	0.50	1.52e-09
WT 6	2.94e-27	2.93e-26	1.00	9.82e-25	1.93e-05
E-cad^{NQ4X}	0.43	0.50	9.82e-25	1.00	1.54e-07
xit	7.51e-08	1.52e-09	1.93e-05	1.54e-07	1.00
